# Peripheral Processing Facilitates Optic Flow-Based Depth Perception

**DOI:** 10.3389/fncom.2016.00111

**Published:** 2016-10-21

**Authors:** Jinglin Li, Jens P. Lindemann, Martin Egelhaaf

**Affiliations:** Department of Neurobiology and Center of Excellence Cognitive Interaction Technology, Bielefeld UniversityBielefeld, Germany

**Keywords:** spatial vision, optic flow, brightness adaptation, photoreceptors, LMCs, computational modeling, fly, natural environments

## Abstract

Flying insects, such as flies or bees, rely on consistent information regarding the depth structure of the environment when performing their flight maneuvers in cluttered natural environments. These behaviors include avoiding collisions, approaching targets or spatial navigation. Insects are thought to obtain depth information visually from the retinal image displacements (“optic flow”) during translational ego-motion. Optic flow in the insect visual system is processed by a mechanism that can be modeled by correlation-type elementary motion detectors (EMDs). However, it is still an open question how spatial information can be extracted reliably from the responses of the highly contrast- and pattern-dependent EMD responses, especially if the vast range of light intensities encountered in natural environments is taken into account. This question will be addressed here by systematically modeling the peripheral visual system of flies, including various adaptive mechanisms. Different model variants of the peripheral visual system were stimulated with image sequences that mimic the panoramic visual input during translational ego-motion in various natural environments, and the resulting peripheral signals were fed into an array of EMDs. We characterized the influence of each peripheral computational unit on the representation of spatial information in the EMD responses. Our model simulations reveal that information about the overall light level needs to be eliminated from the EMD input as is accomplished under light-adapted conditions in the insect peripheral visual system. The response characteristics of large monopolar cells (LMCs) resemble that of a band-pass filter, which reduces the contrast dependency of EMDs strongly, effectively enhancing the representation of the nearness of objects and, especially, of their contours. We furthermore show that local brightness adaptation of photoreceptors allows for spatial vision under a wide range of dynamic light conditions.

## 1. Introduction

Animals have to acquire and process sensory information about the spatial layout of the environment to be able to navigate successfully in cluttered environments. Depth information can be obtained by processing binocular cues from the retinal images of the two eyes. However, small fast-moving animals, such as many insects, are limited in binocular depth vision to very short ranges, because of the small distance between their eyes (Collett and Harkness, 1982). In order to obtain information about the spatial layout of the environment, these animals can use the visual image displacements on the retina (“optic flow”) induced during ego-motion (Egelhaaf et al., 2012). When moving through an environment, the optic flow does not only depend on the speed and direction of ego-motion, but also on the distance to objects in the environment. During translational ego-motion, near objects appear to move faster than far objects. By contrast, during rotational movements, the optic flow depends solely on the velocity of ego-motion and, thus, is independent of the distance to objects (Koenderink, 1986; Vaina et al., 2004; Strübbe et al., 2015). Many insect species, and also some birds, shape their flight and gaze strategies based on this principle. Praying mantises and locusts, for example, may generate translational optic flow by peering movements of the body and the head to estimate distances (Collett, 1978; Sobel, 1990; Kral and Poteser, 1997). Flies and bees, and also birds, employ saccadic flight and gaze strategies which largely separate translational from rotational ego-motion; these facilitate spatial vision during the intersaccadic intervals between the brief and rapid saccadic turns (Schilstra and Hateren, 1999; Hateren and Schilstra, 1999; Eckmeier et al., 2008; Mronz and Lehmann, 2008; Boeddeker et al., 2010; Braun et al., 2010, 2012; Egelhaaf et al., 2012, 2014; Kress et al., 2015; Muijres et al., 2015).

The optic flow and, thus, spatial information, is not available explicitly at the input of the visual system, but needs to be computed from the changing brightness values on the retina. Given the fact that the brightness in natural scenes may vary not only tremendously on a wide range of time scales, i.e., between day and night, but also more rapidly when moving, for instance, through bushland with shady and sunlit patches, it is by no means obvious how consistent optic flow-based information about the depth structure of natural environments can be extracted. Although much is known about optic flow processing, this ecologically highly relevant issue has not been investigated before and, therefore, will be analyzed in this paper by computational modeling.

Optic flow information is computed in a series of processing steps. Photoreceptors in the retina transduce light intensity into graded membrane potential changes. Since photoreceptors have to cope with a wide range of light intensities, while their operating range is limited, they adjust their sensitivity dynamically to the current brightness level by adaptive mechanisms (Laughlin and Hardie, 1978; Laughlin, 1981; Juusola, 1995). Photoreceptors can be modeled, in the simplest approximation, by saturation-like nonlinearities (Naka and Rushton, 1966; Lipetz, 1971; Shoemaker et al., 2005; Schwegmann et al., 2014a) that mimic their steady-state responses to light stimuli. Various types of temporal low-pass filters have been included in the models to account for time-dependent features of photoreceptor responses (James, 1992; Lindemann, 2005). More elaborate model versions rely on optimized filter kernels and divisive feedback to match both steady-state and dynamic photoreceptor responses at a wide range of light levels (Juusola et al., 1995) and even naturalistic brightness fluctuations (van Hateren and Snippe, 2001). The photoreceptors are synaptically connected to the large monopolar cells (LMCs) in the first visual neuropil, the lamina. It is a distinguishing feature of LMCs that they eliminate the mean from the overall luminance of the input to enhance luminance changes (van Hateren, 1992a, 1993, Laughlin, 1994; Juusola, 1995; Juusola et al., 1996; Brenner et al., 2000). However, they perform in this way only when it is sufficiently bright (light-adapted state). The LMCs also tend to represent the absolute brightness level, i.e., have response characteristics of a temporal low-pass filter when it is relatively dark (dark-adapted state) (Juusola, 1995; van Hateren, 1997). For light-adapted conditions, LMCs can be modeled very parsimoniously by temporal band-pass filters (Lindemann, 2005; Shoemaker et al., 2005; Schwegmann et al., 2014a). Several filter kernels of LMCs in a more elaborated model version were optimized separately for each brightness level according to physiological LMC responses (Juusola et al., 1995).

Explicit motion information is extracted from the LMC output locally in the second visual neuropil, the medulla (Egelhaaf and Borst, 1993; Egelhaaf, 2006; Borst et al., 2010; Borst, 2014; Silies et al., 2014). The underlying neural circuitry could be unraveled in unprecedented detail during recent years by combining genetic, behavioral, and electrophysiological approaches (Reiff et al., 2010; Clark et al., 2011; Behnia et al., 2014; Mauss et al., 2014; Tuthill et al., 2014; Ammer et al., 2015; Fisher et al., 2015). The overall performance of these neural circuits of motion detection can be accounted for quite well by a model circuit, the correlation-type elementary motion detector (EMD), that lumps the complex neural circuitry into a relatively simple computational structure (Reichardt et al., 1961; Borst and Egelhaaf, 1989, 1993; Egelhaaf and Borst, 1993; Borst, 2000; Clifford and Ibbotson, 2002). The EMDs have been shown to mimic many response properties of motion-sensitive neurons in an insect's visual motion pathway and form a well established concept for explaining the processing of optic flow in the brains of invertebrates (Hassenstein and Reichardt, 1956), as well as vertebrates (Anderson and Burr, 1985; Santen and Sperling, 1985; Clifford and Ibbotson, 2002). However, EMDs do not encode the retinal velocity and, thus, optic flow unambiguously: Their output does not only depend exclusively on velocity, but also on the pattern properties of a moving stimulus, such as its contrast and spatial frequency content. Hence, nearness information cannot be extracted unambiguously from EMD responses (Egelhaaf and Borst, 1993; Dror et al., 2001; Rajesh et al., 2005; Straw et al., 2008; Meyer et al., 2011; O'Carroll et al., 2011; Hennig and Egelhaaf, 2012). A recent model study suggested that EMD responses to translational optic flow resemble a representation of the contrast-weighted nearness (CwN) to objects in the environment or, in other words, of the contours of nearby objects (Schwegmann et al., 2014a,b). This conclusion, however, needs to be qualified, because it does not take the dynamic rescaling of local light intensities by the visual system via adaptive processes into account, potentially influencing the extraction of depth information from EMD responses.

In the present study, therefore, we assess the functional consequences of the different processing stages in the peripheral visual system and their adaptive features on the representation of spatial information at the output of the motion detection system when stimulated with naturalistic translational image flow (Schwegmann et al., 2014a). We will show that nearby contours in natural cluttered environments are represented robustly by local motion detectors, irrespective of a wide range of adaptive parameter changes in the peripheral visual system, as long as the average brightness level is largely eliminated from the input signals. This allows for the extraction of depth information and detection of nearby contours from the optic flow generated during self-motion under a wide range of light levels. These findings may generalize to other biological and technical visual systems based on correlation-type movement detection, because these are also limited in the sensor coding range and resolution (Harrison and Koch, 1999; Köhler et al., 2009; Plett et al., 2012; Meyer et al., 2016).

## 2. Methods

### 2.1. Visual motion pathway models

**Figure 1:**
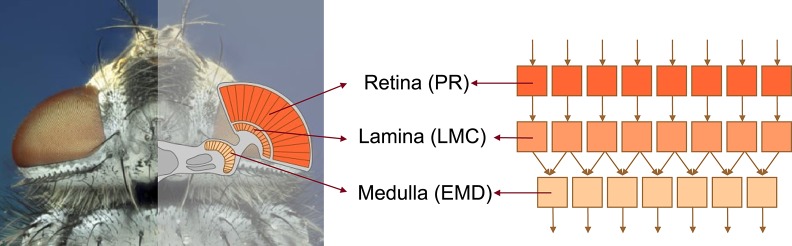
**Schematic illustration of the structure of the insect visual motion pathway using the example of the blowfly**. The visual motion pathway of the blowfly comprises retinotopically arranged successive processing stages of retina (containing photoreceptors, PRs), lamina (containing large monopolar cells, LMCs), and medulla (location of elementary motion detection, EMD) (**left** subfigure), which are correspondingly modeled by model units of *PR, LMC, EMD*, respectively (**right** subfigure).

The model of the visual motion pathway of insects, such as flies, is composed of successive retinotopically organized stages reminiscent of their biological counterparts: the retina (photoreceptors, PRs), the lamina (LMCs), and the medulla (local motion detection circuits that are conventionally modeled by EMDs) (Figure 1).

**Figure 2:**
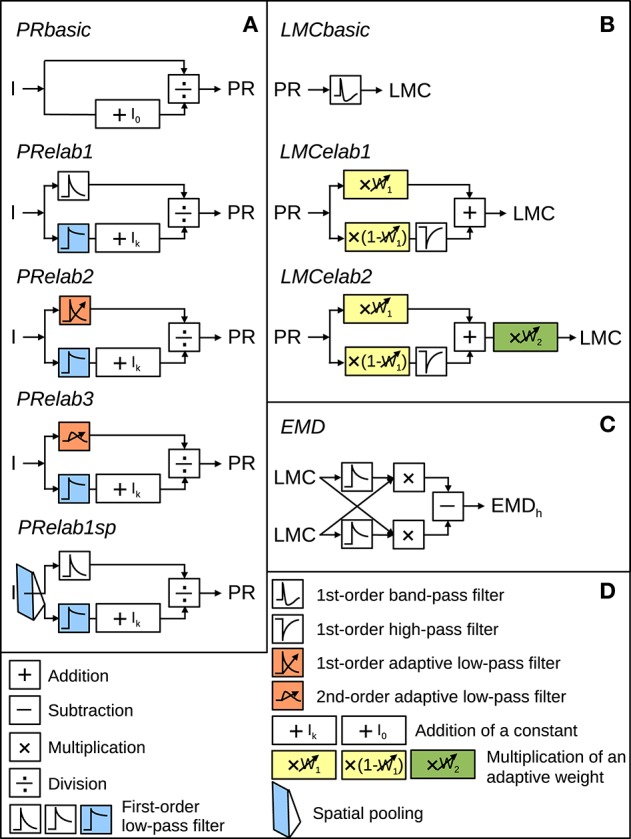
**Model variants of components of the insect visual motion pathway. (A)** Photoreceptor model variants (*PRbasic* to *PRelab1sp*) for signal processing in the retina. **(B)** Large monopolar cells model variants (*LMCbasic* to *LMCelab2*) for signal processing in the lamina. **(C)** Correlation-type EMD. **(D)** Description of symbols used in **(A–C)** with units accounting for different adaptive response features being color-coded. *I* stands for input intensity and *PR, LMC*, and **EMD**_**h**_ for the model responses of PRs, LMCs, and the EMD array in a horizontal direction, respectively. (See Section 2 for detailed model descriptions and Appendix A for the parameter setting of the model variants above).

The models of the processing stages preceding motion detection were configured to account for the main response features of their biological counterpart, as published in earlier studies (Laughlin and Hardie, 1978; Juusola, 1995), in a potentially most parsimonious way. Various versions of photoreceptor and LMC models were tested to understand which response features of the peripheral visual system are essential for robust spatial vision. They differed in their complexity by including incrementally further computational elaborations, such as temporal filters, parallel branches with different properties, and/or adaptive elements (Figure 2). These model versions of the peripheral visual system serve as input to an array of EMDs (Figure 2, Schwegmann et al., 2014a).

The model parameters were determined via systematic variation of parameters and by choosing parameter combinations that capture the main response features of photoreceptors or LMCs qualitatively. The parameters selected for each model variant are summarized in Appendix A. All simulations were done in time steps of 1 ms.

#### 2.1.1. Photoreceptor models

The input-output transformation of photoreceptors was elaborated incrementally by the following steps: A static saturation-like nonlinear transformation was modeled as a basic photoreceptor model (Figure 2, *PRbasic*), according to

(1) $$\begin{array}{l} PR=\frac{I}{I+I_{0}} \end{array}$$

to capture the steady-state response of photoreceptors (Naka and Rushton, 1966; Lipetz, 1971; Shoemaker et al., 2005; Schwegmann et al., 2014a). In Equation (1), *PR* represents the photoreceptor response, *I* the input light intensity and *I*_0_ a constant determining the light intensity corresponding to half-maximum response. We introduced a model component as in

(2) $$\begin{array}{l} PR=\frac{PRlp1\left(I\right)}{PRlp2\left(I\right)+I_{k}} \end{array}$$

in the first elaboration step (Figure 2, *PRelab1*) to describe the adaptive temporal response profile of photoreceptors and the adaptive shift of the intensity-response function. The input intensities are in one signal branch, low-pass filtered with a small time constant (*PRlp1 (I)*), leading to a signal that follows the time course of intensity fluctuations and in a parallel signal branch, low-pass filtered with a large time constant (*PRlp2 (I)*), leading to a signal reflecting the changes of the overall light level on a slower time scale. The latter signal, after adding a constant *I*_*k*_, is used to modify the sensitivity of the photoreceptor (Figure 2, *PRelab1*, color-coded in blue). In the second elaboration step (Figure 2, *PRelab2*), the time constant of the fast temporal low-pass filter (Figure 2, *PRelab2*, color-coded in red, τ_*PRlpa*_) adapts to the current light level (*I*_*level*_) by increasing its cut-off frequency with increasing light level, according to a saturation-like nonlinearity defined by

(3) $$\begin{array}{llll} \; & \tau_{PRlpa}=1/2\left(\tau_{max}-\tau_{min}\right)\left(1-\tanh\left(x_{\tau }\right)\right)+\tau_{min} & \; & \;\\ \; & x_{\tau }=\kappa_{\tau }\log_{10}\left(I_{level}\right)-\mu_{\tau } & \; & \; \end{array}$$

In this equation, τ_*max*_ and τ_*min*_ are the upper and lower boundary of τ_*PRlpa*_, and μ_τ_ and κ_τ_ are the turning point in logarithmic scale and the slope of the saturation-like nonlinear dependency, respectively. Elaboration step 3 (Figure 2, *PRelab3*) took into account that cells cannot respond immediately to a stimulus with a large response amplitude and, thus, replaced the adaptive first-order low-pass filter (*PRlpa*) of *PRelab2* by a second-order one.

#### 2.1.2. LMC models

The output of the final adaptive photoreceptor model (Figure 2, *PRelab3*) was fed into the LMC layer of the visual motion pathway model, which was developed incrementally in a similar way to the photoreceptor models. The basic LMC model (Figure 2, *LMCbasic*) was realized by a temporal band-pass filter (James, 1992; Lindemann, 2005; Shoemaker et al., 2005; Schwegmann et al., 2014a). In this way, the signal component reflecting the mean brightness level is eliminated, as is characteristic of LMC responses under light-adapted conditions. Elaboration step 1 (Figure 2, *LMCelab1*) takes into account that LMCs perform like temporal band-pass filters only under light-adapted conditions, but like low-pass filters under dark-adapted conditions (Juusola, 1995). To account for this feature, the photoreceptor response is split into two branches without overall gain change, i.e., the sum of the weights in the two branches is one. These two branches are unfiltered and high-pass filtered, respectively, to account for the low-pass and band-pass filtered component of LMC responses. The weight of the unfiltered branch (*w*_1_, Figure 2, *LMCelab1*, color-coded in yellow) increases adaptively with decreasing photoreceptor response input. The dependency of *w*_1_ on the photoreceptor response is described in

(4) $$\begin{array}{lll} w_{1} & =1/2\left(w_{1max}-w_{1min}\right)\left(1-\tanh\left(x_{w1}\right)\right)+w_{1min}\; & \;\\ x_{w1} & =\kappa_{w1}\log_{10}\left(PR\right)-\mu_{w1}\; & \; \end{array}$$

in which *w*_1*max*_ and *w*_1*min*_ are the upper and lower boundaries of *w*_1_, and μ_*w*1_ and κ_*w*1_ are constants describing the turning point in logarithmic scale and the slope of the saturation-like nonlinear dependency, respectively. Elaboration step 2 (Figure 2, *LMCelab2*) mimics the adaptive modulation of the contrast gain of LMC responses (Juusola, 1995); therefore, a weighting factor (*w*_2_, Figure 2, *LMCelab2*, color-coded in green) was introduced which increases adaptively with decreasing light level. Similar to *w*_1_, the dependency of *w*_2_ on the light level (*I*_*level*_) is described by

(5) $$\begin{array}{lll} w_{2} & =1/2\left(w_{2max}-w_{2min}\right)\left(1-\tanh\left(x_{w2}\right)\right)+w_{2min}\; & \;\\ x_{w2} & =\kappa_{w2}\log_{10}\left(I_{level}\right)-\mu_{w2}\; & \; \end{array}$$

in which *w*_2*max*_ and *w*_2*min*_ are the upper and lower boundaries of *w*_2_, and μ_*w*2_ and κ_*w*2_ are constants describing the turning point in logarithmic scale and the slope of the saturation-like nonlinear dependency, respectively.

#### 2.1.3. Retinal area and time scale of peripheral adaptation

Facing a vast dynamic range of light intensities, visual systems have to adjust their operating range according to current light conditions. The current light level is obtained within a certain retinal area and on a certain time scale. Since, to the best of our knowledge, no experimental data on the retinal range of brightness adaptation are available, the development and validation of the adaptive peripheral models of photoreceptors and LMCs had to be based on cell responses to point stimuli under a set of adapted conditions (Laughlin and Hardie, 1978; Juusola, 1995) (see below for explanation of the stimuli). Therefore, we did not make an *a priori* assumption regarding the retinal area and time scale of adaptation, but varied both adaptive parameters systematically to assess their potential functional role.

Specifically, the current light level (*I*_*level*_) in Equations (3) and (5), that is used to adjust the time constant of the low-pass filter of photoreceptors (Figure 2, color-coded orange) and the weight of contrast gain of LMCs (Figure 2, color-coded green) are defined as follows. We varied the retinal area of adaptation by determining the overall light level (*I*_*level*_) within a two-dimensional (2D) Gaussian window (1 × 1 pixel^2^ to 71 × 71 pixel^2^, with each pixel covering 1.25° of the visual field corresponding to the angular distance between adjacent ommatidia). The time scale of adaptation was varied by defining the current light level as average light intensity within a certain time period (0–500 ms) before the respective signal is employed for tuning the adaptive parameters. However, since we found that the variation of the retinal area and time scale of these two adaptive processes does not affect the performance in spatial vision much (data not shown), we set the adaptation to be local and instantaneous in the further analyses, i.e., *I*_*level*_ = *I*, for simplification.

With respect to the adaptive shift of the sigmoidal input response characteristic of the photoreceptors (Figure 2, *PRelab1*), the output of the lower signal branch (Figure 2, *PRelab1*, color-coded blue) reflects the current overall light level, which shifts the input-response function of the photoreceptor via a divisive nonlinear operation. The time course of this adaptive process is constrained by physiological data, whereas the retinal range is not. We, therefore, varied the retinal area of adaptation by changing the half-width of the 2D Gaussian filtering applied to the intensity input to this branch, as described above (lower branch of photoreceptor input in Figure 2, *PRelab1sp*). Since varying the retinal area of brightness adaptation of photoreceptors reveals an advantage of local adaptation (1 × 1 pixel^2^) for spatial vision (**Figure 11**), local adaptation is, therefore, used as a default setting.

#### 2.1.4. EMD model

The output of these model variants of the peripheral visual system is fed into the next processing stage consisting of correlation-type EMDs (Reichardt et al., 1961; Borst and Egelhaaf, 1989, 1993; Egelhaaf and Borst, 1993; Borst, 2000; Clifford and Ibbotson, 2002) (Figure 2, *EMD*). Each EMD is composed of two mirror-symmetrical subunits, the half-detectors. In each half-detector, the delayed signal originating from one LMC unit is multiplied by the undelayed signal originating in the neighboring LMC unit. This is carried out in parallel at each location in the visual field for horizontal and for vertical neighbors. Subtraction of the signals of corresponding mirror-symmetric half-detectors leads to the EMD responses to vertical (**EMD**_**v**_) and horizontal motion (**EMD**_**h**_), respectively. The sign of the movement detector output indicates the direction of the motion detected. The motion energy is computed by taking the norm of the motion vector given by the combination of the responses of a pair of the **EMD**_**h**_ and the **EMD**_**v**_ at a given location (*x*,*y*) of the visual field (Schwegmann et al., 2014a):

(6) $$\begin{array}{l} EMD=\sqrt{EMD_{h}^{2}+EMD_{v}^{2}} \end{array}$$

### 2.2. System-analytical stimuli and electrophysiological data

**Figure 3:**
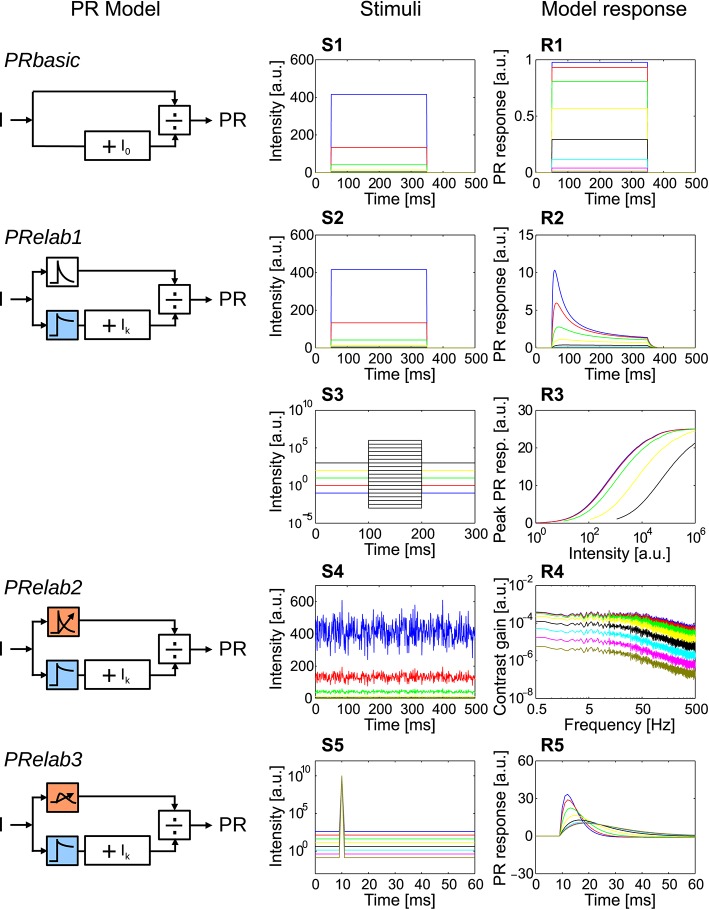
**Response properties included in each step elaboration of the photoreceptor model. (*PRbasic*–*PRelab3*)** Photoreceptor model variants. **(S1–S5)** Time course of point stimuli that were fed into the PR models (each color represents a light condition). **(R1–R5)** Model responses to the corresponding point stimuli. **(S1,R1)** Intensity steps and step responses of *PRbasic*. **(S2,R2)** Intensity steps and step responses of *PRelab1*. **(S3)** Sets of intensity steps under each light level to determine input-response characteristics for each light level by plotting the peak response to each intensity step, and **(R3)** input-response characteristics of *PRelab1* for each adaptive light level. **(S4)** Pseudo-random intensity fluctuations, and **(R4)** frequency dependence of contrast gain obtained based on fast Fourier transformation of the responses of *PRelab2*. **(S5,R5)** Impulse stimuli and impulse responses of *PRelab3* (See Section 2, Figure 2, and Appendix A for the model discription and parameters; and Appendix B for the descriptions of parameters of point stimuli and corresponding response analysis).

**Figure 4:**
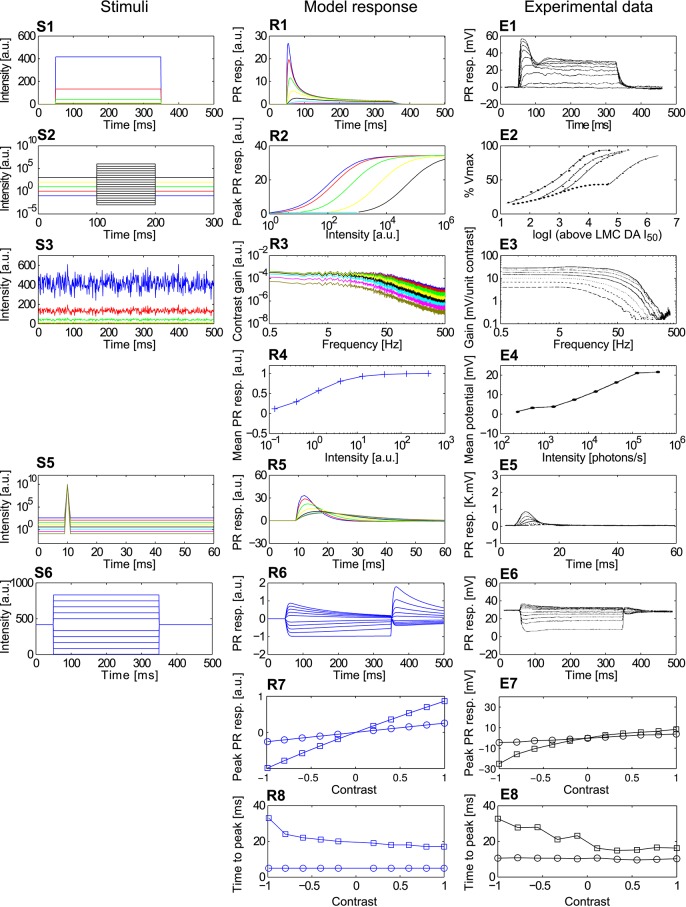
**Comparison of the adaptive photoreceptor model ***PRelab3*** with fly photoreceptor responses. (S1–S6)** Point stimuli used in the model and electrophysiological analyses. **(R1–R8)** Corresponding responses of *PRelab3* model and **(E1–E8)** of blowfly photoreceptors (data from Laughlin and Hardie, 1978; Juusola, 1995). **(S1,R1,E1)** Intensity steps and step responses. **(S2)** Sets of intensity steps and **(R2,E2)** corresponding intensity-response curves of *PRelab3* and photoreceptors for different background intensities. **(S3)** Pseudo-random light intensity fluctuations, **(R3,E3)** corresponding frequency dependence of the contrast gain, and **(R4,E4)** average responses of *PRelab3* and photoreceptors to pseudo-random fluctuations over time for various background intensity levels. **(S5,R5,E5)** Impulse stimuli and impulse responses. **(S6,R6,E6)** Long contrast steps under light-adapted conditions, and corresponding model and cell responses. **(R7,E7)** Peak responses to long (□) and short (○) contrast steps under light-adapted conditions **(R8,E8)** and corresponding time-to-peak for model and photoreceptor responses. (See Appendix B for the descriptions of parameters of point stimuli and corresponding response analysis).

**Figure 5:**
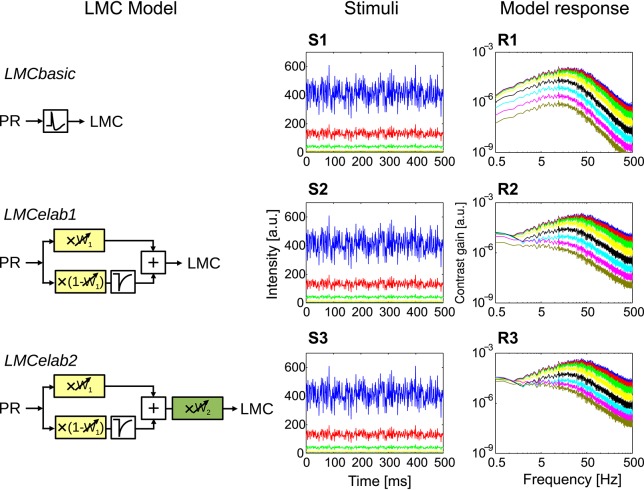
**Response properties of different LMC model variants. (*LMCbasic*–*LMCelab2*)** The LMC model variants, using the responses of *PRelab3* as input. **(S1–S3)** Point stimuli of pseudo-random fluctuations and **(R1–R3)** frequency dependence of the contrast gain of LMC model responses. (See Section 2, Figure 2, and Appendix A for the model description and parameters; and Appendix B for the descriptions of parameters of point stimuli and corresponding response analysis).

**Figure 6:**
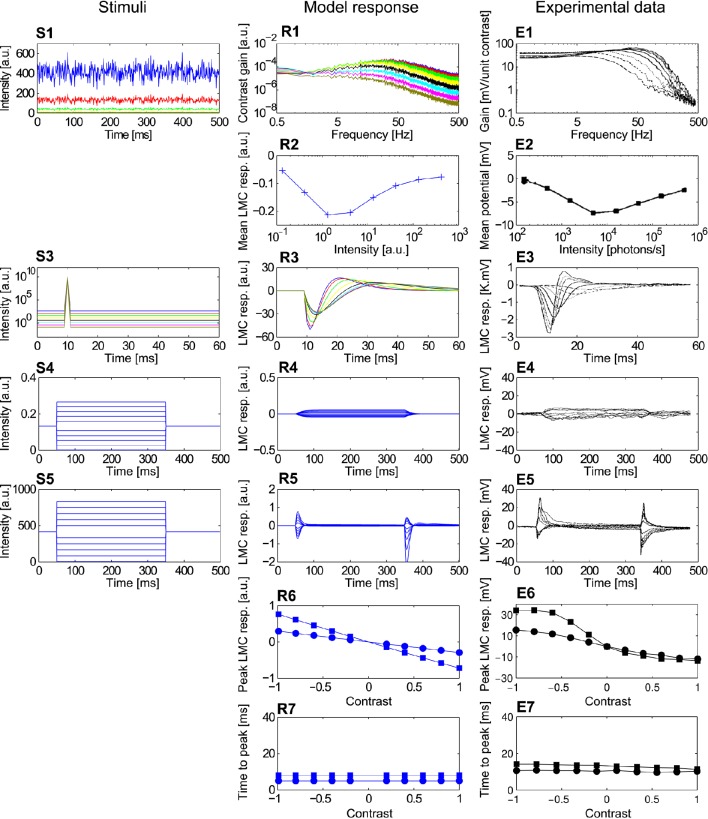
**Comparison of the ***LMCelab2*** model with the corresponding fly LMC responses. (S1–S5)** Point stimuli used for the model and electrophysiological analyses. **(R1–R7)** Corresponding responses of *PRelab3-LMCelab2* model and **(E1–E7)** LMCs (data from Laughlin and Hardie, 1978; Juusola, 1995). **(S1)** Pseudo-random light intensity fluctuations, **(R1,E1)** frequency dependence of contrast gain and **(R2,E2)** average responses of *LMCeslab2* and LMC over time to the pseudo-random fluctuations for various background light levels. **(S3,R3,E3)** Impulse stimuli and corresponding model and cell responses. **(S4,R4,E4)** Long contrast steps under dark-adapted conditions and corresponding model and cell responses. **(S5,R5,E5)** Long contrast steps under light-adapted conditions and corresponding model and cell responses. **(R6,E6)** Peak responses to long (■) and short (•) contrast steps under light-adapted conditions and **(R7,E7)** corresponding time-to-peak for model and LMC responses. (See Appendix B for the descriptions of parameters of point stimuli and corresponding response analysis).

The models of photoreceptors and LMCs are developed and validated based on previous electrophysiological data (Laughlin and Hardie, 1978; Juusola, 1995). In these studies, responses of photoreceptors and LMCs were recorded using a set of experimenter-designed point stimuli. These stimuli included intensity steps, contrast steps and pseudo-random brightness fluctuations, i.e., a constant background light level over time superimposed with white noise intensity fluctuations with a standard deviation proportional to the background light level. Each type of stimulus was presented in the previous experimental analysis at various background brightness levels to capture the major response features of photoreceptors and LMCs under a wide range of adaptive conditions (Juusola, 1995). We applied the same stimuli when developing our model of the peripheral visual system. Additionally, we included impulse stimuli in our model analysis, although impulse responses in the experimental study were computed from the responses to pseudo-random intensity fluctuations. Furthermore, a set of step stimuli with a wide range of intensities for each adaptive light level was presented in our model. The sensitivity curve for each light level was derived by plotting the peak responses to each intensity step for each adaptive light level. This set of stimuli was similar to those used in another experimental study of photoreceptor and LMC responses (Laughlin and Hardie, 1978). These stimuli are shown in Figures 3–6, and the parameters for all of the stimuli above are summarized in Appendix B.

### 2.3. Naturalistic stimuli

In order to understand the role of adaptive peripheral processing for spatial vision based on motion information, we used stimuli similar to the retinal input that an insect experiences in natural environments, i.e., image sequences mimicking the retinal projections of the outside world on the eyes during translational ego-motion in natural surroundings (Schwegmann et al., 2014a). These image sequences were acquired in the following way: A high dynamic range camera was mounted at a height of 0.5 m on a motor-driven linear feed in natural environments and moved along a linear track for 1 m. The camera took one panoramic image per cm distance with the help of a panoramic hyperboloidal mirror. The pixel values were proportional to the light intensity in the green spectral range (arbitrary units). This procedure was repeated in 37 different natural environments. The image sequences obtained in this way were interpolated 10-fold to mimic the visual input during continuous translational motion at 1 m/s. A panoramic rectangular lattice with square pixels was obtained by unwrapping the panoramic images obtained with the hyperpoloidal mirror system projections. The image sequences obtained in this way were filtered and resized with 2D spatial Gaussian filters to simulate the spatial filtering property of insect photoreceptors, assuming an acceptance angle of 1.64° and interommatidial angular distance of 1.25°, thereby image sequences with panoramic rectangular lattice of 73 × 289 pixel^2^ were obtained (for details, see Schwegmann et al., 2014a and the data in Schwegmann et al., 2014c published online).

In order to analyze the model performance under various light conditions (**Figures 10–12**), we artificially rescaled the input intensity of these image sequences into different intensity ranges with upper and lower boundaries. The rescaling is calculated according to

(7) $$\begin{array}{rcl} \log_{10}\left(I_{scale}\right)\; & = & \;\frac{\log_{10}\left(I\right)-\log_{10}\left(min\left(I\right)\right)}{\log_{10}\left(max\left(I\right)\right)-\log_{10}\left(min\left(I\right)\right)}\left(B_{up}-B_{low}\right)\\ \;+\;B_{low} \end{array}$$

in which *I* is the intensity of the original image sequences, *I*_*scale*_ is the respective rescaled intensity value, and *B*_*up*_ and *B*_*low*_ are the upper and lower boundaries of the rescaled intensity range in logarithmic scale, respectively.

### 2.4. Assessment of model performance

In order to assess the model performance in representing behaviorally relevant environmental parameters, such as local brightness contrast, nearness of objects, and the contours of nearby objects (contrast weighted nearness, CwN) at the level of arrays of motion detectors, we adopted the same measure of correlation between the response profile of arrays of EMDs and these environmental parameters as in Schwegmann et al. (2014a). Firstly, the local contrast around one pixel was calculated according to

(8) $$\begin{array}{c} \begin{array}{l} C_{\left(x,y\right)}\;=\;\frac{std\left(I_{X,Y}\right)}{mean\left(I_{X,Y}\right)}\;\\ \;X=\left[x-1,x,x+1\right],\;Y=\left[y-1,y,y+1\right] \end{array} \end{array}$$

Local contrast maps were obtained by applying this process to each pixel. Secondly, a distance map of the environment (the distance of the corresponding points in the environment to the moving camera) was obtained from the optic flow fields. These were determined using a computer vision algorithm for image velocity estimation (Lucas and Kanade, 1981) in the implementation of the Matlab computer vision system toolbox (Schwegmann et al., 2014a). Nearness maps were calculated as the reciprocal of the distance maps. Thirdly, the CwN maps were defined by the product of the local contrast maps and the nearness maps. Finally, we calculated the logarithmic correlations between the local contrast, the nearness and the CwN obtained for the frame in the middle of a translational segment and the EMD responses of the frames for time-shifts between 0 and 50 ms. The time-shift at which the correlation coefficient was largest (usually around 20 ms) was used for evaluating the model performance. These calculations were performed for image sequences and EMD responses obtained in 37 different environments. The time-shift for the best correlation varies slightly between model variants (18.0 ± 3.4 ms), environmental parameters (17.5 ± 5.6 ms) and also image sequences (17.5 ± 10.2 ms) if taking the model variants that contain both PR and LMC into account.

## 3. Results

### 3.1. Modeling an adaptive peripheral visual system

#### 3.1.1. Development and assessment of photoreceptor model versions

In order to obtain a photoreceptor model, we attributed specific experimentally determined response properties of photoreceptors to several computational processing units and integrated these incrementally into the version of the photoreceptor model that qualitatively captures most response features of photoreceptors (Figure 3) (See Figure 2, Appendix A, and Section 2 for model description and parameter setting; and Appendix B for description and parameter setting of point stimuli and response analysis). A saturation-like nonlinearity was introduced as the most basic photoreceptor model (Figure 3, *PRbasic*). The responses of *PRbasic* (Figure 3R1) to intensity steps (Figure 3S1) reflect the static nonlinear transformation of light intensity into steady-state responses, as is characteristic of photoreceptors. The time-dependent decay of the photoreceptor response to intensity steps (Figure 3R2) was modeled by low-pass filtering the two parallel signal branches subserving each sampling point in space with different time constants before the signal filtered with the smaller time constant is divided by the signal with the larger one (Equation 2, Figure 3, *PRelab1*). Apart from accounting for the decay in the response amplitude, this model version already accounts for the adaptive shift of the input-response function with increasing light intensity (Figures 3R3,S3). This feature is striking, since *PRelab1* does not contain any genuine adaptive elements. Thus, this photoreceptor model can operate under a wide range of light conditions without being saturated. In the second elaboration step (Figure 3, *PRelab2*), a genuine adaptive element was included by modeling the corner frequency of the low-pass filter to shift to lower frequencies with a decreasing light level (Figures 3S4,R4). In the final elaboration (Figure 3, *PRelab3*), the time course of the model response was further adjusted by replacing the first-order low-pass filter by a second-order one, because the photoreceptor responses to impulse stimuli do not reach their maximum instantaneously, as is predicted when using a first-order low-pass filter (Figures 3S5,R5).

The responses of the photoreceptor model *PRelab3* to various types of stimuli are compared in Figure 4 to electrophysiological data on fly photoreceptors published previously (Laughlin and Hardie, 1978; Juusola, 1995). (1) The step response of both model and biological photoreceptors show fast response decay within approximately 50 ms before reaching their steady state (Figures 4R1,E1). (2) The shift in the stimulus-response characteristic toward larger brightness values with increasing overall brightness (Figure 4R2) reproduces qualitatively that of the corresponding electrophysiological data (Figure 4E2). (3) The shift of the cutoff frequency of the frequency dependence of the contrast gain of the model responses to random brightness fluctuations (Figure 4R3) is shifted to lower values with decreasing overall brightness, similar to that of fly photoreceptors (Figure 4E3). This is accomplished by decreasing the time constant of the adaptive low-pass filter with increasing light level from 2.3 ms for the light-adapted condition to 9 ms for the dark-adapted condition. (4) The mean model and electrophysiological responses to random brightness fluctuations (Figure 4S3) increase both with increasing mean brightness until they reach a saturation level (Figures 4R4,E4). (5) The peak of the impulse responses of both the model and the photoreceptor is increasingly delayed and reduced in amplitude with decreasing overall brightness (Figures 4R5,E5). (6) The model and photoreceptor responses to contrast steps are asymmetric with respect to stimulus polarity: In particular, the responses to brightness increases are more transient and reach a smaller steady-state response amplitude than the responses to brightness decreases (Figures 4R6,E6). However, the amplitudes of the off-responses of the model are larger than those of the fly photoreceptors. Furthermore, the peak responses and time-to-peak were calculated for the long (Figure 4S6) and short contrast steps (same as Figure 4S6, but with 2 ms step stimuli). (7) The slightly smaller peak model response for a positive long contrast step than for a negative long contrast step is reflected in a similar asymmetry in photoreceptor responses, although it is less prominent (Figures 4R7,E7, □). (8) The time-to-peak of both model and photoreceptor responses to contrast steps of either polarity is rather independent of the contrast value of short stimuli (○), whereas it increases with the brightness level for long stimuli (□) with increasing negative contrast (Figures 4R8,E8).

#### 3.1.2. Development and assessment of LMC model versions

The incrementally developed versions of LMC models and their main response features are illustrated in Figure 5 (See Figure 2, Appendix A, and Section 2 for model description and parameter setting; and Appendix B for description and parameter setting of point stimuli and response analysis). As a basic model, a first-order temporal band-pass filter was introduced (Figure 5, *LMCbasic*) to mimic the most distinguishing feature of LMCs under light-adapted conditions, i.e., the elimination of the average brightness level from the incoming photoreceptor signals. The contrast gain of *LMCbasic* responses to pseudo-random brightness fluctuations (Figures 5S1,R1) depends on frequency in a way similar to a band-pass. Since biological LMCs tend to perform like a low-pass filter representing the average brightness at very low light levels, this feature was considered in the first elaboration of the LMC model (Figure 5, *LMCelab1*) by summing up the weighted high-pass filtered and unfiltered output signals of the photoreceptor model. The sum of weights of the two signals is kept constant (i.e., one), with the weight of the unfiltered signal increasing with decreasing brightness. The frequency dependence of the contrast gain of this model version, as determined by random brightness fluctuations, changes smoothly from a characteristic more similar to that of a low-pass to a characteristic similar to that of a band-pass when the system shifts from a dark-adapted to a light-adapted state (Figure 5R2). A weighting factor in *LMCelab2* (Figure 5, *LMCelab2*), which increases with decreasing brightness, is multiplied by the output signal of *LMCelab1* to take into account the fact that the contrast gain of biological LMCs stays within a rather narrow range, even for a wide range of light levels, with only a slightly higher gain under medium brightness levels (Figure 5R3).

The responses of *LMCelab2* are compared with the corresponding LMC responses, (Juusola, 1995). (1) The band-pass characteristic of both model and LMCs changes to a low-pass-like characteristic with decreasing overall light level; in parallel, the corner frequencies shift to smaller values (Figures 6R1,E1). (2) The mean response amplitude of model and LMCs to random brightness fluctuations is largest for intermediate overall brightness levels, while they are relatively small under dark or bright conditions (note that LMC responses to brightness increases are negative) (Figures 6R2,E2). Apart from the responses which were used for model development, *LMCelab2* also reproduces, without any further adjustment, other qualitative features observed in the electrophysiological data. (3) The peak of impulse responses of both the model and LMCs is increasingly reduced and delayed, and the overshoot of the impulse responses is also increasingly reduced with decreasing light level (Figures 6R3,E3). (4, 5) Both *LMCelab2* and LMCs perform like a low-pass filter under dark-adapted conditions (Figures 6R4,E4) and like a band-pass filter under light-adapted conditions (Figures 6R5,E5). However, the model reveals stronger off-responses to negative contrasts. (6, 7) Again, the peak responses and time-to-peak were calculated for the long (Figure 6S5) and short contrast steps (same as Figure 6S5, but with 2 ms step stimuli). The peak responses of both model and LMCs (Figures 6R6,E6) to short contrast steps (•) of either polarity increase with increasing amplitude less than those to long contrast steps (■). The time-to-peak of both the model and LMCs is rather independent of the contrast value and polarity (Figures 6R7,E7), although the model responses are several milliseconds faster than their biological counterparts. This feature may be a consequence of the latencies in the biological system not being implemented by this model.

### 3.2. Impact of peripheral processing on representing environmental parameters by motion detectors

#### 3.2.1. Functional significance of the individual peripheral processing units in spatial vision

**Figure 7:**
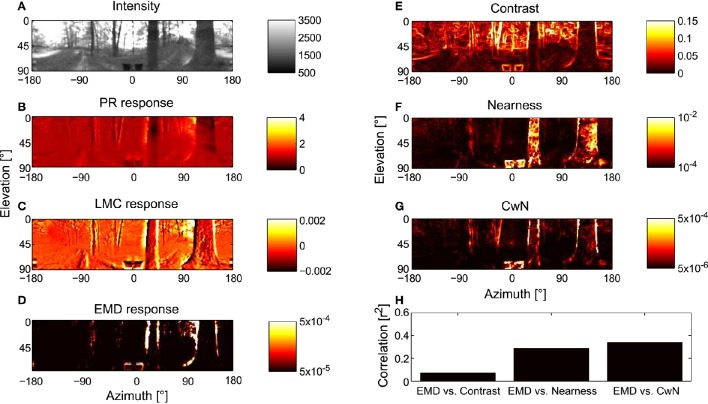
**Representation of moving natural scenery and environmental parameters in the visual motion pathway. (A)** Retinal image taken at the center of a translational sequence of camera motion through a natural environment. Intensity values are proportional to those in the green spectral range (arbitrary units). Corresponding representation at successive processing stages of the visual motion pathway, **(B)** array of PRs (*PRelab1*), **(C)** array of LMCs (*LMCbasic*), and **(D)** arrays of EMDs (see Figure 2) shown as map of motion energies. **(E–G)** Spatial maps of environmental parameters for evaluation of model performance: **(E)** local contrast map, **(F)** nearness map, and **(G)** contrast-weighted nearness (CwN) map (see Section 2). **(H)** Correlation between the response profile of EMD and the different environmental parameters. The correlation shown for the environmental parameters in the middle of the translation sequence and, because of stimulus-response phase shifts, the EMD responds about 20 ms later (see Section 2).

In order to assess how signal processing in the peripheral visual system, including light adaptation, affects the representation of environmental information by the motion detection system during translational locomotion in cluttered environments, we combined models of photoreceptors and LMCs as developed above with correlation-type EMDs and stimulated this model of the visual motion pathway with image sequences that were based on translational movements of a panoramic camera system through a variety of natural environments (see Section 2). Model variant *PRelab1-LMCbasic-EMD* in Figure 7, for example, was stimulated with image sequences mimicking the visual input during translational flight in a forest. The panoramic visual stimuli (Figure 7A) and the response profiles of simulated 2D arrays of photoreceptors (Figure 7B), of LMCs (Figure 7C), and of motion energies provided by arrays of EMDs (Figure 7D), as taken during the middle of the translational sequence, are illustrated. How well environmental cues are represented at the level of motion detectors is further assessed by calculating pixelwise the correlation (Figure 7H) between the response profile of the EMDs (Figure 7D) and the contrast (Figure 7E), the nearness (Figure 7F) and the CwN maps, respectively (Figure 7G) (see Section 2). In accordance with Schwegmann et al. (2014a), the high correlation with CwN suggests, at least in this example, that arrays of EMDs represent predominantly contours of nearby objects.

**Figure 8:**
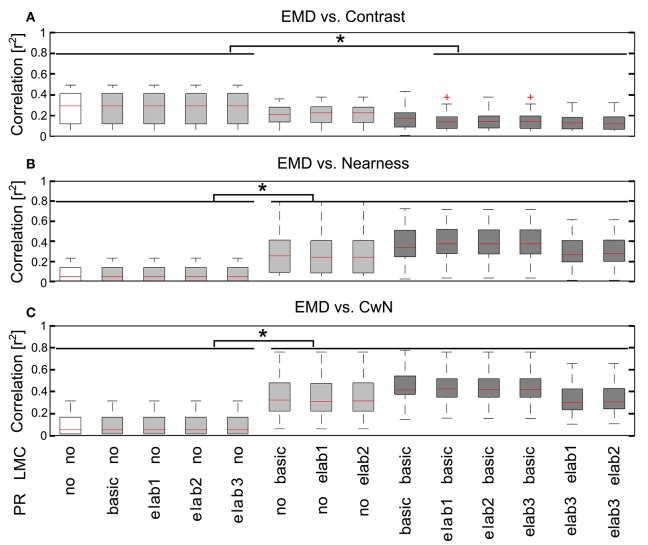
**Consequences of the different peripheral processing units on information representation at the EMD output**. Correlations between the motion energy profile of EMDs and the local contrast map **(A)**, the nearness map **(B)**, and the CwN map **(C)** for all 37 natural environments tested illustrated by box plots (red line, median; box, 25–75 percentile; red cross, outlier). Correlations were determined for 14 model variants, as indicated below the figure panels [*PR*: PR model variants (see Figure 2), in which *no* means without any processing at the PR stage; *LMC*: LMC model variants (see Figure 2), *no* means without LMC stage signal processing. White: EMD without any peripheral processing; gray: EMDs with different PR models, but without LMC processing (four gray boxes on the left), and EMDs with different LMC models, but without PR processing (three gray boxes on the right); black: EMDs with different combinations PR and LMC models]. Asterisks indicate a significant difference (*p* < 0.05, Kruskal–Wallis test with *post hoc* multiple comparison).

**Figure 9:**
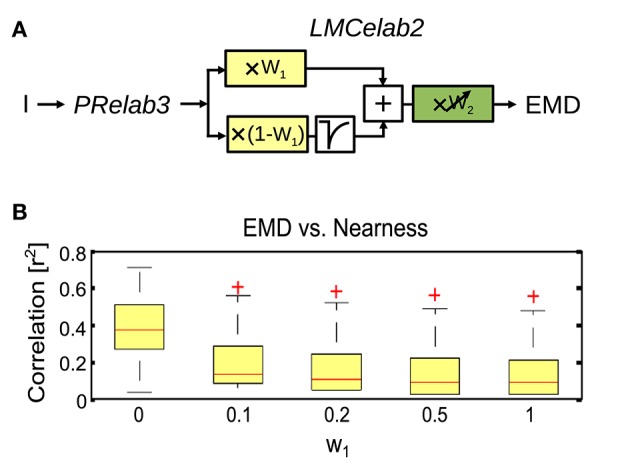
**Impact of mean brightness components in the LMC response on the extraction of spatial information by EMDs. (A)** Variant of visual motion pathway model *PRelab3-LMCelab2-EMD* (see Figure 2), in which the relative weight (*w*_1_) of the unfiltered and high-pass filtered branch of the LMC model is not adaptive, but is kept constant at different levels. **(B)** Correlation between EMD response profile and the nearness map for all 37 naturalistic stimuli summarized in the boxplot (red line, median; box, 25–75 percentile; red crosses, outliers), and compared between variants with different weight (*w*_1_) for the unfiltered branch, which contains the information about the mean brightness level.

In order to draw general conclusions valid for a wide range of natural environments and to understand the role of each peripheral processing unit, we applied the same performance assessment to 37 different natural environments and 14 different model variants (Figure 8). We applied a Kruskal–Wallis test and *post hoc* multiple comparison to test whether the performance of different model variants are significantly different (*p* < 0.05). Without any peripheral processing (Figure 8, white boxes), the EMD responses depend greatly on local contrast and are hardly correlated to the nearness or CwN. The other basic or adaptive photoreceptor model versions alone, i.e., without LMC processing, do not change this situation much (Figure 8, first four gray boxes). However, if the basic or adaptive LMC models are fed directly by the brightness signals without the preceding photoreceptor model, the contrast-dependence of the EMD responses is reduced and the correlation between EMD responses and nearness, as well as the CwN is enhanced significantly. Again, this conclusion is independent of whether a basic or an elaborated version of the LMC model is employed (Figure 8, last three gray boxes). If the LMCs are fed by any of the photoreceptor models, the contrast dependency of the EMD responses further decreases and the correlation with nearness and CwN further improves (Figure 8, black boxes). The model performance is robust against any various adaptive elaboration of the photoreceptors (Figure 8, first four black boxes). However, nearness or CwN are represented best for the combined photoreceptor and LMC model versions if the basic LMC versions rather than the adaptive ones are used (Figure 8, last two black boxes). The slight reduction in performance when introducing an adaptive elaboration of the LMC model (Figure 8, last two black boxes) has its reason in the representation of the overall brightness by LMC if it is not very bright, because part of the images may be dark in many environments. Figure 9 illustrates how the performance in nearness extraction decreases with an increasing representation of the mean brightness level (i.e., fixed weight of low-pass filtered signal component in the response) (Figure 9B).

To sum up, motion-based extraction of spatial information is robust against a variety of adaptive changes in the photoreceptor model (Figure 8, first four black boxes) and LMC model, as long as the average brightness information is largely eliminated from the EMD input as is characteristic of the light adapted insect peripheral visual system (Figure 8, last two black boxes, and Figure 9). Essentially, the elimination of the signal component reflecting the mean brightness of the scenery in the retinal input signals shifts a primarily contrast-based representation of natural environments at the output of the arrays of local movement detectors during translational ego-motion to a representation that reflects its spatial structure (Figure 8, white and gray boxes, and Figure 9). These conclusions lead to our most parsimonious model version for optic flow-based spatial vision: *PRelab1-LMCbasic-EMD* (See Supplementary Movie S1 for the performance comparison between pure EMD and the suggested model variant *PRelab1-LMCbasic-EMD*). The following analysis is, therefore, based on this model variant.

#### 3.2.2. *PRelab1* enables robust spatial vision under a vast range of light conditions

**Figure 10:**
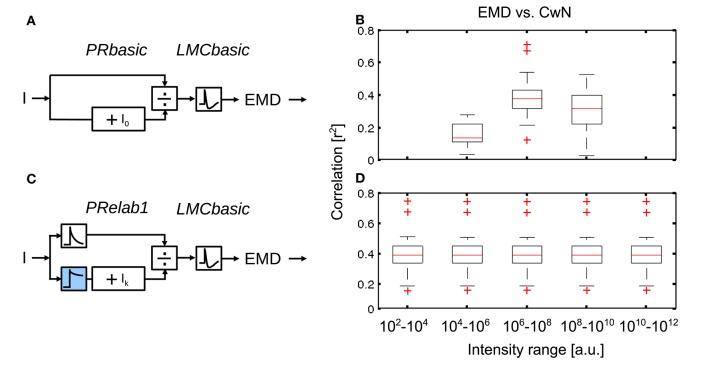
**Role of brightness adaptation for spatial vision over a vast range of light intensity. (A)** Non-adaptive visual motion pathway model *PRbasic-LMCbasic-EMD*, with *I*_0_ = 10^7^, and **(C)** adaptive visual motion pathway model *PRelab1-LMCbasic-EMD* (see Figure 2 and Appendix A). **(B,D)** Correlation between EMD response profile and CwN map for all 37 naturalistic stimuli summarized in the boxplot under various artificially generated light conditions, compared between model variants in **(A,C)**.

The brightness-dependent shift of the input-response function of photoreceptors (Figure 3R3) allows for the encoding of contrast under a vast range of light conditions. In order to further assess the role of this adaptive shift of the photoreceptors' input-response function for spatial vision, we rescaled the same set of naturalistic image sequences artificially to various light levels covering ten decades of intensities (see Equation 7 in Section 2) and compared the performance of non-adaptive *PRbasic-LMCbasic-EMD* with that of the adaptive *PRelab1-LMCbasic-EMD* model variants (Figure 10). Since photoreceptors have a limited membrane potential range to encode brightness signals and their responses are affected by noise, their capacity to resolve brightness changes is limited. We mimicked the consequences of this limited resolution of photoreceptors in a very crude way by discretizing the photoreceptor output into 12 bit (2^12^) levels between the maximal and the minimal photoreceptor responses. Simulation results reveal that the EMD response profile of the non-adaptive model variant (Figure 10A), which is most sensitive at light levels of 10^7^ (i.e., *I*_0_ = 10^7^), represents the nearby contours of objects sufficiently well only in this brightness range 10^6^−10^8^, but largely fails under higher or lower light levels (Figure 10B). By contrast, the adaptive model (Figure 10C) represents the contours of nearby objects robustly over the entire range of light conditions examined (Figure 10D). It should be noted that, although the performance of the model version *PRelab1* (see Equation 2 and Figure 2) tested appears to be adaptive, it contains no adaptive parameters nor any feedback of the current response level or average input intensity. Rather, its excellent performance in representing spatial information over a wide range of brightness conditions is based exclusively on dividing the signals of two parallel pathways reflecting light intensity on a fast and a slow time scale, respectively (See Supplementary Movie S2 for the performance of model variant *PRelab1-LMCbasic-EMD* under light conditions vary by 8 decades). Since we could show that the adaptive model variant allows for robust spatial vision under a vast range of light conditions, even with restricted coding resolution, the further simulations of this study are done, for the sake of simplicity, without the limitation in coding resolution.

#### 3.2.3. Impact of the retinal area of brightness adaptation on optic flow-based spatial vision

**Figure 11:**
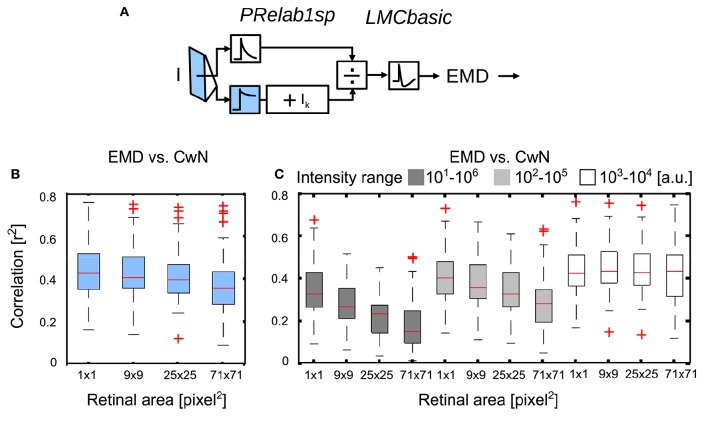
**Effect of the retinal area of brightness adaptation of photoreceptor on spatial vision. (A)** Variant of visual motion pathway model *PRelab1sp-LMCbasic-EMD* (see Figure 2 and Appendix A). Signal input to the lower branch of the adaptive PR model (blue), which reflects the current light level used for adaptation, was spatially pooled with 2D spatial Gaussian filter with different half-widths (1 × 1, 9 × 9, 25 × 25, 71 × 71 pixel^2^, with each pixel corresponding to 1.25° of the visual field). **(B)** Correlation between response profile of EMDs and CwN under various retinal areas of adaptation. **(C)** The same as **(B)**, only the range of input intensity was artificially rescaled (Equation 7) to cover five decades (black), three decades (gray), one decade (white), respectively (intensity values in arbitrary units).

No experimental data are available concerning the retinal area that determines the adaptive state of photoreceptors. Since we did not want to make *a priori* assumptions about this important aspect of brightness adaptation, we varied this area in our model simulations systematically to understand its role from the perspective of spatial vision. As the signal branch filtered by *PRlpf2* (Figure 11A, color-coded blue) is responsible for reflecting the current light condition in this model variant, we varied the retinal area of the input to this branch by 2D Gaussian filtering of the retinal intensity profile (from 1 × 1 pixel^2^ to 71 × 71 pixel^2^, with each pixel corresponding to 1.25° of visual field; Figure 11B; See section 2.1.3). With an increasing retinal area of adaptation, the performance of arrays of motion detectors to represent nearby contours decreases slightly (Figure 11B). In a further set of simulations, we rescaled the input intensities, according to Equation (7), to either five, three or one decades, i.e., the intensity values were rescaled to 10–10^6^ (Figure 11C, black), 10^2^–10^5^ (Figure 11C, gray), or 10^3^–10^4^ (Figure 11C, white), respectively. With the increasing range of input intensity (white to black), the performance decreased strongly with an increasing retinal area of adaptation. These results suggest local adaption of photoreceptors to be advantageous. The wider the intensity range within a given scenery, the more relevant it is for photoreceptors to be locally adaptive in order to maintain the performance of EMDs in spatial vision (See Supplementary Movie S3 for the performance comparison between local adaptation and global adaptation under a wide intensity range).

#### 3.2.4. Time scale of brightness adaptation and its relevance in spatial vision during free flight

The speed of fast brightness adaptation is not challenged by the day-night cycle, but rather by the dynamics of locomotion. Taking the flight dynamics of blowflies or bees as an example, fast brightness adaptation should allow the visual motion vision pathway to extract spatial information within tens of milliseconds. This fast time scale is demanded given the characteristic flight and gaze strategies of many insects, which is characterized by up to ten brief and rapid saccadic turns per second interspersed by intersaccadic intervals of mainly translational motion, which may be as short as only few tens of milliseconds (Schilstra and Hateren, 1999; Boeddeker et al., 2015). Since the retinal brightness during flight in natural environments may vary tremendously between consecutive intersaccadic intervals as a consequence of saccadic turns, brightness adaptation should take place during saccadic turns and only during the very first phase of intersaccadic intervals to allow the motion vision pathway to extract spatial information from intersaccadic optic flow.

**Figure 12:**
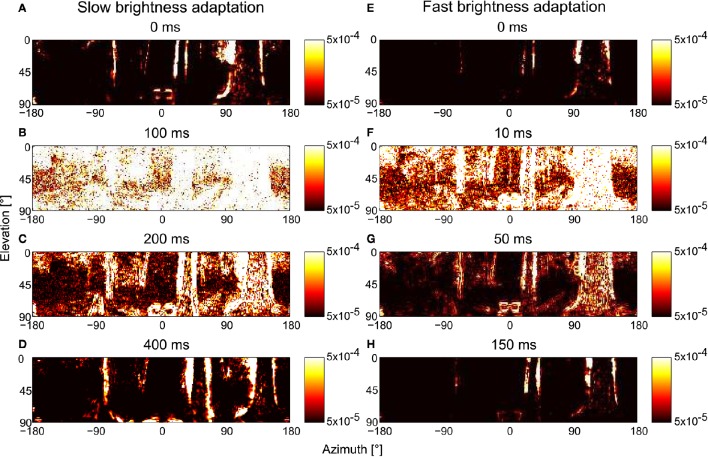
**Consequences of the time scale of brightness adaptation for representing spatial information by arrays of EMDs after an abrupt change in light intensity during translational motion in a forest**. The model simulations are based on the *PRelab1-LMCbasic-EMD* version. For *t* ≤ 0, the brightness values are artificially rescaled to 10^2^–10^4^, and for *t* >0, to 10^4^–10^6^. **(A–D)** EMD response profiles with default parameter setting, i.e., τ_*PRlp*1_ = 9 ms, τ_*PRlp*2_ = 250 ms; and **(E–H)** with parameter settings that allow for faster brightness adaptation, i.e., τ_*PRlp*1_ = 2 ms, τ_*PRlp*2_ = 20 ms (see Figure 2 and Appendix A).

We changed the overall brightness of the scenery during the translational trajectories to assess the adaption speed and, thus, the time after a rapid brightness change required by the motion vision system to recover its ability to provide spatial information. The light intensities during the first 300 ms of the trajectory is rescaled to the range of 10^2^–10^4^, and the remaining approximately 700 ms is rescaled to the range of 10^4^–10^6^. The rescaled image sequence was fed into the *PRelab1-LMCbasic-EMD* model, and the restoration of spatial information at the arrays of motion detectors over time after the abrupt brightness change is illustrated (Figure 12). With the default parameter setting (i.e., the parameters determined according to the physiological data in Juusola, 1995 and used so far in our analysis, Figures 12A–D), the motion detectors are almost saturated by the input signal for roughly 100 ms after the brightness change (Figure 12B). The representation of the spatial information is largely recovered after 200 ms (Figure 12C), and totally recovered about 400 ms after the brightness shift (Figures 12A,D). This speed of adaptation is much slower than that required by the flight dynamics of a fly, i.e., the duration of translational intersaccadic flight (see above). Note, however, that the saccade frequencies were measured under spatially constrained conditions and under light conditions without systematic intensity changes between saccades (Schilstra and Hateren, 1999; Boeddeker et al., 2015). Thus, intersaccadic intervals in free flight in spatially less constrained natural environments might be longer allowing for more time to adapt spatial vision to abrupt brightness changes. Moreover, even the brightest light levels used in the electrophysiological experiments (approximately 400 cd/m^2^, Juusola, 1995) that were employed to tune our model parameters were much darker than sunlit natural environments. Therefore, the response transients and the speed of adaption under bright outdoor conditions could be faster than those obtained under laboratory conditions. Faster brightness adaptation can also be achieved in our model by adjusting τ_*PRlp*1_ and τ_*PRlp*2_ of *PRelab1* (Figures 12E–H). With this parameter setting, the spatial information is restored to a large extent within 50 ms after an abrupt brightness change (Figure 12G) and is already fully restored within 150 ms (Figure 12H). This time scale of adaptation matches the requirements of insect flight dynamics. The restoration of spatial vision after an abrupt decrease in brightness works in a similar way, but on a somewhat slower time scale due to the asymmetries in the PR and LMC model responses to light decrements and increments, respectively (See Supplementary Movie S4 for the restoration of spatial vision after an abrupt change in brightness with default and fast-adaptive parameter settings).

## 4. Discussion

One challenge of animal visual systems is to extract behaviorally relevant information consistently in natural environments, irrespective of the strong variations of light levels. It has not yet been understood how this is accomplished regarding the spatial vision of aerial insects, which is assumed to be based on optic flow information during translational flight segments (Egelhaaf et al., 2012, 2014). One problem is the contrast and pattern dependence of biological motion detectors that compute optic flow information in insects (Egelhaaf and Borst, 1993; Dror et al., 2001; Rajesh et al., 2005; Straw et al., 2008; Meyer et al., 2011; O'Carroll et al., 2011; Hennig and Egelhaaf, 2012). Furthermore, the range of physical parameters characterizing the retinal images, such as brightness, often extends over several orders of magnitude, while the operating range of photoreceptors and neurons is much more limited. Therefore, our aim was to understand how information regarding the spatial layout of natural environments can be consistently extracted under a wide range of dynamic brightness conditions. In order to tackle these questions, we modeled the visual motion pathway of insects systematically based on physiological data and analyzed the influence of the different processing stages on the representation of spatial information at the level of motion detectors. Eliminating the average brightness information from the photoreceptor signals, as is characteristic of neurons in the first visual neuropil under light-adapted conditions, is found to be indispensable for reducing the contrast dependence of motion detector responses and to facilitate the representation of spatial information at the motion detection layer. The representation of spatial information has been found to be very robust against various adaptive mechanisms in the peripheral visual system, as long as the average brightness information is eliminated from the input to the motion detectors. The adaptive shift of the input-response characteristic of photoreceptors especially allows the system to extract spatial information consistently under a wide range of dynamic brightness conditions.

### 4.1. Retinal range and time scale of brightness adaptation in the peripheral visual system

The light intensities in natural environments can vary by up to nine to ten orders of magnitude, whereas the response range of photoreceptors is limited to tens of millivolts, which corresponds to only three to four log units of input light intensity (flies: Laughlin and Hardie, 1978; vertebrates: Normann and Perlman, 1979). However, an unsaturated and consistent representation of the environments may be required over a much larger brightness range, demanding photoreceptors to adapt to the current brightness level for successful visually guided behavior.

Furthermore, the dynamics of the intensity fluctuations in the retinal image are not only influenced by the overall brightness level changing in the slow time course of the day-night cycle, but also on much faster time scales that are shaped by the dynamics of the animals specific locomotion behavior, as well as the spatial distribution of light intensity over a given scenery. In order to facilitate the extraction of behaviorally relevant information, for instance, several aerial insect species, and also birds, actively shape their visual input while exploring their surroundings by a saccadic flight and gaze strategy (Schilstra and Hateren, 1999; Hateren and Schilstra, 1999; Eckmeier et al., 2008; Mronz and Lehmann, 2008; Boeddeker et al., 2010, 2015; Braun et al., 2010, 2012; Kern et al., 2012; Kress et al., 2015; Muijres et al., 2015). Since the duration of translational flight between saccadic turns usually lasts for only some tens of milliseconds (Egelhaaf et al., 2012), at least the fast component of adaptation to dynamic intensity fluctuations should be fast enough to be useful.

Moreover, adaptation should not cover large parts of the visual field in the same way, because global adaptation would hardly prevent saturation in part of the visual field if different parts of the scenery contain contrasting details at both very light and very dark brightness levels. Hence, localized adaptation is demanded. This, however, is only possible at the expense of losing global contrast information. On the other hand, adaptation should also not be too local and fast, in order to maintain relevant contrast information, especially when looking at stationary or slowly changing scenes. Therefore, the time scale and retinal area of adaptation should match constraints imposed by the statistics of the natural environment and the specific dynamics of locomotion. Laughlin (1981) pointed out that brightness and contrast adaptation based on a transformation function matching the accumulated probability density distribution of intensity leads to the most efficient use of the operating range of photoreceptors. However, it is not entirely clear on which temporal and spatial scale the statistics of the intensity distribution should be considered.

Among all the adaptive mechanisms of the peripheral visual system modeled in this study (Figure 2, color-coded), the adaptive shift of the sigmoidal input-response characteristic of photoreceptors is most obviously functionally relevant (Figure 2, color-coded blue). This adaptive feature has already been modeled before and implemented in bio-inspired hardware (Delbrück and Mead, 1994; Beaudot, 1996; Shoemaker et al., 2005; Mafrica et al., 2015). This is accomplished by shifting the point of inflection of the underlying sigmoidal Lipetz (alternatively Naka-Rushton) transformation (Beaudot, 1996; Shoemaker et al., 2005; Mafrica et al., 2015) or by a gain modulation in an amplified feedback loop (Delbrück and Mead, 1994; Mafrica et al., 2015). Adaptation in these models is based either on using the average input intensity to adjust the adaptive parameter directly (Shoemaker et al., 2005) or by feeding back the current response level (Delbrück and Mead, 1994). The retinal area of adaptation is modeled by taking the brightness either globally (frame-wise) (Shoemaker et al., 2005; Schwegmann et al., 2014a) or locally (pixel-wise) into account (Delbrück and Mead, 1994; Mafrica et al., 2015). The time scale of adaptation is either instantaneous (Shoemaker et al., 2005) or modeled by the time constant of the feedback loop (Delbrück and Mead, 1994).

Our adaptive photoreceptor model (*PRelab1* as the simplest case) is based on a different principle: The signal in the fast branch (see Figure 2) follows the current intensity fluctuations quite well, while the slower branch mediates brightness information on a slower time scale and adjusts the sensitivity of the fast branch by a divisive nonlinearity. In this way, a seemingly adaptive performance (Figures 3R3, 10D) is realized without any feedback or parameter changes. The times cale of this adaptive feature of our model is constrained by physiological data obtained from blowfly photoreceptors (Juusola, 1995). However, the restoration of spatial information after abrupt brightness changes is slower than that required for spatial vision during translational intersaccadic flight of insects, at least if the relatively short intersaccadic time intervals observed under spatially contrained conditions are taken as a reference (Schilstra and Hateren, 1999; Boeddeker et al., 2015). It might well be the case that the saccade frequency is lower in spatially less constrained natural environments and, thus, intersaccadic intervals long, in order to allow for the recovery of spatial vision. On the other hand, the experiments (Juusola, 1995) which were used to adjust our model time constants were conducted under relatively dark conditions due to technical limitations. It is, thus, conceivable that the time constants that determine the speed of adaption might be faster under the much brighter outdoor conditions. Subsequently, a much faster recovery of spatial information after abrupt brightness changes could be observed in our model simulation that meets even the free flight dynamics measured under spatially constrained conditions (Figure 12).

Since the experiments used to calibrate our model of the peripheral insect visual system are based on point stimuli under various brightness conditions, the retinal area of adaptation could not be constrained by these data. Therefore, we systematically varied the retinal area of adaptation and analyzed its impact on the representation of spatial information by motion detectors. By varying the pooling range of the input to the slow branch of the photoreceptor model, we could show its influence on the spatial information conveyed by the motion vision system. The more localized the adaptation is, the better the visual system performs in representing spatial information at the motion detector output, especially when it is facing the challenge of a wide range of input intensity values (Figure 11C).

### 4.2. Functional significance of adaptive peripheral processing on spatial vision

Information processing by the peripheral visual system, including adaptive processes, has usually been conceptualized within an information-theoretical framework, i.e., with the perspective of transmitting as much information about the environment as possible by a channel with limited information capacity and reliability. The temporal band-pass filtering by LMCs, for instance, has been suggested to be beneficial for reducing redundancy and increasing coding efficiency (Brenner et al., 2000). Accordingly, the adaptive peripheral processing has been concluded to maximize transmitted information, given the restricted coding range of neurons (Laughlin, 1981; Brenner et al., 2000), to maximize the signal-to-noise ratio, given noisy peripheral channels and noisy environmental conditions (van Hateren, 1992b), and to save energy without loss of information (Rasumov et al., 2011).

In this study, we employed a complementary approach which looks at peripheral information processing and adaptation from the perspective of its role in representing environmental features at the output of the visual motion pathway, such as contours of near objects (Schwegmann et al., 2014a), which may play a functional role in controlling visually guided behavior. Pursuing a similar concept, Dror et al. (2001) have shown that some peripheral preprocessing units, such as spatial blurring, temporal high-pass filtering and response saturation, can increase the quality of velocity coding by EMDs of different natural images rotating at various velocities. Here, we made a step toward even more realistic conditions and studied the consequences of various peripheral processing mechanisms on the representation of spatial information by arrays of EMDs during translational ego-motion in cluttered environments.

We could show that elimination of the average light level by the peripheral visual system is not only useful for reducing redundancy and enhancing intensity changes, but also indispensable for the representation of spatial information by the motion detector output (Figure 8). Under realistic conditions of translational motion in natural environments, the response profile of EMDs without any peripheral processing depends so much on contrast that it is barely able to represent any depth information (Figure 8). Elimination of the average brightness level by the band-pass filtering characteristic of LMCs reduces the contrast dependence and enhances the representation of the environmental depth structure by the EMD responses. We additionally show that a saturation-like nonlinearity of the photoreceptor model further improves this performance by the compression of the response (Figure 8). These results are consistent with the findings of Dror et al. (2001). Moreover, we could show that motion-based spatial vision during translational ego-motion in cluttered environments is rather robust against light adaptation in the peripheral visual system (Figure 8), as long as the average brightness level is eliminated from the photoreceptor signal (Figure 9), which allows for a consistent extraction of spatial information under a vast range of brightness conditions (Figure 10).

A specific feature of peripheral processing which is shared both by vertebrates and insects is the separation of on-off pathways in the input lines of EMDs (Sanes and Zipursky, 2010; Eichner et al., 2011; Silies et al., 2013; Behnia et al., 2014; Ammer et al., 2015; Leonhardt et al., 2016). This has not been included in this study for the sake of simplicity. The relevance of on- and off-pathways, of non-linearities in the peripheral visual system as well as of multiple neural input to output cells of the motion detection circuit in representing contours of different contrast polarity could recently be shown with motion stimuli containing correlations higher than second order (Takemura et al., 2001; Fitzgerald et al., 2011; Clark et al., 2014; Fitzgerald and Clark, 2015). To what extent the responses to higher-order motion stimuli are affected by adaptive processes, as are characteristic of the peripheral visual system and the mechanism of motion detection itself, has not yet been analyzed. The potential functional consequences of part of the aforementioned architectural elaborations of the motion detection circuit with regard to extracting optic flow-based spatial information will be considered in the context of motion adaptation in a study on which we are currently working.

Since correlation-type motion detection in its various elaborations appears to be a general principle in both insects (Hassenstein and Reichardt, 1956) and vertebrates (Anderson and Burr, 1985; Santen and Sperling, 1985; Clifford and Ibbotson, 2002) and is also used in some artificial visual systems (Harrison and Koch, 1999; Köhler et al., 2009; Plett et al., 2012), our results suggest a general way of reducing the contrast dependency and enhancing the representation of spatial information by this kind of motion detector when operating in natural environments. Since brightness adaptation is also a common requirement for both biological and artificial visual systems that operate in natural environments, our findings may generalize to a wider range of vision systems.

## Author contributions

JL, JPL, and ME conceptualized and designed the project. JL developed the models and performed model simulations. JL, JPL, and ME wrote the paper.

## Funding

This work was supported by the Cluster of Excellence Cognitive Interaction Technology “CITEC” (EXC 277) at Bielefeld University, which is funded by the Deutsche Forschungsgemeinschaft (DFG). The publication fee was funded by the Open Access Publication Funds of Bielefeld University.

### Conflict of interest statement

The authors declare that the research was conducted in the absence of any commercial or financial relationships that could be construed as a potential conflict of interest.

## Appendix

### A.parameters for model variants in Figure 2

*PRbasic*: *I*_0_ is a constant reflecting the inflection point of saturation-like nonlinearity realized by *PRbasic*, indicating the intensity corresponding to half maximal photoreceptor response. In Figure 8, *I*_0_ is the average intensity of the image sequence for each environment, and in Figure 10, it is the mid-point of the intensity range investigated (10^7^); *PRelab1*: as a default setting, the time constant for the low-pass filter in the upper branch (τ_*PRlp*1_) is 9 ms, time constant for the low-pass filter in the lower branch (τ_*PRlp*2_) is 250 ms, and *I*_*k*_ = 10. As a faster adaptive setting, τ_*PRlp*1_ = 2 ms, τ_*PRlp*2_ = 20 ms, and *I*_*k*_ = 10 (Figure 12); *PRelab2*: the same as *PRelab1*, except that τ_*PRlpa*_ has a nonlinear dependency on the light level (*I*_*level*_) as in Equation (3), in which *I*_*level*_ represents the light levels: 416, 133, 41.6, 13.2, 4.16, 1.33, 0.416, and 0.133 in arbitrary units, τ_*max*_ = 9 ms, τ_*min*_ = 2 ms, μ_τ_ = 1, κ_τ_ = 1; *PRelab3*: the same as *PRelab2*, except duplication of τ_*PRlpa*_ into second-order adaptive low-pass filter; *LMCbasic*: τ_*LMClp*_ = 8 ms, τ_*LMChp*_ = 5 ms; *LMCelab1*: τ_*LMChp*_ = 5 ms , *w*_1_ has a nonlinear dependency on photoreceptor response, as in Equation (4), in which *w*_1*max*_ = 0.75, *w*_1*min*_ = 0.25, μ_*w*1_ = −1.5, κ_*w*1_ = 1.5; *LMCelab2*: the same as *LMCelab1*, except that *w*_2_ has nonlinear dependency on the light level, as in Equation (5), in which *w*_2*max*_ = 6, *w*_2*min*_ = 2, μ_*w*2_ = 1, κ_*w*2_ = 1; EMD: τ_*EMDlp*_ = 40 ms, calculation of motion energy according to Equation (6).

### B.parameters for point stimuli in Figures 3–6 and corresponding response analysis

Intensity steps: intensity steps changing from dark to eight logarithmically distributed light levels (*I*_*level*_), with *t*_*start*_ = 50 ms, *t*_*step*_ = 300 ms, *t*_*end*_ = 150 ms, *I*_*level*_: 416, 133, 41.6, 13.2, 4.16, 1.33, 0.416, and 0.133 in arbitrary units (the same below). A set of intensity steps: a set of intensity steps (*I*_*step*_) under each light level (*I*_*bg*_) to determine input-response characteristics for each light level by plotting the peak response to each intensity step, in which *t*_*start*_ = 100 ms, *t*_*step*_ = 100 ms, *t*_*end*_ = 100 ms, *I*_*bg*_: 0.1, 1, 10, 100, and 1000 in arbitrary units, and *I*_*step*_: 181 logarithmically equally distributed intensity values between 10^−3^ and 10^6^ in arbitrary units. Pseudo-random fluctuations: eight logarithmically equally distributed constant light levels (*I*_*level*_) over time are superimposed with white noise with a certain contrast (*c* = 0.32). The frequency dependence of contrast gain to these stimuli is obtained by fast Fourier transforming of the model or cell responses and dividing them by the contrast (Hamming window of length *L* = 2^17^ was applied before fast Fourier transformation, and the power spectrum of *n* = 100 segments was averaged). Impulse stimuli: brief intensity impulse under each light level (*I*_*level*_), with *t*_*start*_ = 10 ms, *t*_*impulse*_ = 1 ms, *t*_*end*_ = 50 ms, *I*_*impulse*_ = 10^10^ in arbitrary units. Long contrast steps under light-adapted condition: long stepwise increment or decrement of intensity with various contrasts (*cs*), starting from light-adapted condition (*I*_*light*_), in which *t*_*start*_ = 50 ms, *t*_*contrast*_ = 300 ms, *t*_*end*_ = 150 ms, *I*_*light*_ = 416 in arbitrary units, *cs* varying from −1 to 1 in 0.2 intervals without 0 (the same below). Short contrast steps under light-adapted condition: similar to the last stimuli, only with a shorter duration of step contrast, i.e., *t*_*contrast*_ = 2 ms. Long contrast steps under dark-adapted conditions: similar to the stimuli—long contrast steps under light-adapted conditions, only starting from a dark adapted condition, i.e., *I*_*dark*_ = 0.13 in arbitrary units.

## References

[B1] Ammer G. Leonhardt A. Bahl A. Dickson B. J. Borst A. (2015). Functional specialization of neural input elements to the drosophila on motion detector. Curr. Biol. 25, 2247–2253. 10.1016/j.cub.2015.07.014 26234212

[B2] Anderson S. J. Burr D. C. (1985). Spatial and temporal selectivity of the human motion detection system. Vision Res. 25, 1147–1154. 10.1016/0042-6989(85)90104-X 4071994

[B3] Beaudot W. H. (1996). Sensory coding in the vertebrate retina: towards an adaptive control of visual sensitivity. Network 7, 317–323. 10.1088/0954-898X_7_2_012 16754392

[B4] Behnia R. Clark D. A. Carter A. G. Clandinin T. R. Desplan C. (2014). Processing properties of on and off pathways for drosophila motion detection. Nature 512, 427–430. 10.1038/nature13427 25043016 PMC4243710

[B5] Boeddeker N. Dittmar L. Stürzl W. Egelhaaf M. (2010). The fine structure of honeybee head and body yaw movements in a homing task. Proc. R. Soc. Lond. B 277, 1899–1906. 10.1098/rspb.2009.2326 20147329 PMC2871881

[B6] Boeddeker N. Mertes M. Dittmar L. Egelhaaf M. (2015). Bumblebee homing: the fine structure of head turning movements. PLoS ONE 10:e0135020. 10.1371/journal.pone.0135020 26352836 PMC4564262

[B7] Borst A. (2000). Models of motion detection. Nat. Neurosci. 3, 1168. 10.1038/81435 11127831

[B8] Borst A. (2014). Neural circuits for motion vision in the fly. Cold Spring Harb. Symp. Quant. Biol. 79, 131–139. 10.1101/sqb.2014.79.024695 25527086

[B9] Borst A. Egelhaaf M. (1989). Principles of visual motion detection. Trends Neurosci. 12, 297–306. 10.1016/0166-2236(89)90010-6 2475948

[B10] Borst A. Egelhaaf M. (1993). Detecting visual motion: theory and models, in Visual Motion and Its Role in the Stabilization of Gaze, eds Miles F. Wallman J. (Amsterdam: Elsevier), 3–27. 8420555

[B11] Borst A. Haag J. Reiff D. F. (2010). Fly motion vision. Annu. Rev. Neurosci. 33, 49–70. 10.1146/annurev-neuro-060909-153155 20225934

[B12] Braun E. Dittmar L. Boeddeker N. Egelhaaf M. (2012). Prototypical components of honeybee homing flight behavior depend on the visual appearance of objects surrounding the goal. Front. Behav. Neurosci. 6:1. 10.3389/fnbeh.2012.00001 22279431 PMC3260448

[B13] Braun E. Geurten B. Egelhaaf M. (2010). Identifying prototypical components in behaviour using clustering algorithms. PLoS ONE 5:e9361. 10.1371/journal.pone.0009361 20179763 PMC2825265

[B14] Brenner N. Bialek W. de Ruyter van Steveninck R. (2000). Adaptive rescaling maximizes information transmission. Neuron 26, 695–702. 10.1016/S0896-6273(00)81205-2 10896164

[B15] Clark D. A. Bursztyn L. Horowitz M. A. Schnitzer M. J. Clandinin T. R. (2011). Defining the computational structure of the motion detector in drosophila. Neuron 70, 1165–1177. 10.1016/j.neuron.2011.05.023 21689602 PMC3121538

[B16] Clark D. A. Fitzgerald J. E. Ales J. M. Gohl D. M. Silies M. A. Norcia A. M.. (2014). Flies and humans share a motion estimation strategy that exploits natural scene statistics. Nat. Neurosci. 17, 296–303. 10.1038/nn.3600 24390225 PMC3993001

[B17] Clifford C. W. G. Ibbotson M. R. (2002). Fundamental mechanisms of visual motion detection: models, cells and functions. Prog. Neurobiol. 68, 409–437. 10.1016/S0301-0082(02)00154-5 12576294

[B18] Collett T. (1978). Peering a locust behavior pattern for obtaining motion parallax information. J. Exp. Biol. 76, 237–241.

[B19] Collett T. S. Harkness L. I. K. (1982). Depth vision in animals, in Analysis of Visual Behavior, eds Ingle D. J. Goodale M. A. Mansfield R. J. W. (Cambridge, MA; London: MIT Press), 111–176.

[B20] Delbrück T. Mead C. (1994). Adaptive photoreceptor with wide dynamic range, in Proceedings of IEEE International Symposium on Circuits and Systems-ISCAS'94 (IEEE), 339–342. 10.1109/ISCAS.1994.409266

[B21] Dror R. O. O'Carroll D. C. Laughlin S. B. (2001). Accuracy of velocity estimation by Reichardt correlators. J. Opt. Soc. Am. A 18, 241–252. 10.1364/JOSAA.18.000241 11205969

[B22] Eckmeier D. Geurten B. R. H. Kress D. Mertes M. Kern R. Egelhaaf M.. (2008). Gaze strategy in the free flying zebra finch (*Taeniopygia guttata*). PLoS ONE 3:e3956. 10.1371/journal.pone.0003956 19107185 PMC2600564

[B23] Egelhaaf M. (2006). The neural computation of visual motion information, in Invertebrate Vision, eds Warrant E. Nielsson D. E. (Cambridge: Cambridge University Press), 399–461.

[B24] Egelhaaf M. Boeddeker N. Kern R. Kurtz R. Lindemann J. P. (2012). Spatial vision in insects is facilitated by shaping the dynamics of visual input through behavioral action. Front. Neural Circuits 6:108. 10.3389/fncir.2012.00108 23269913 PMC3526811

[B25] Egelhaaf M. Borst A. (1993). Movement detection in arthropods, in Visual Motion and Its Role in the Stabilization of Gaze, eds Miles F. A. Wallman J. (Amsterdam: Elsevier), 53–77.

[B26] Egelhaaf M. Kern R. Lindemann J. P. (2014). Motion as a source of environmental information: a fresh view on biological motion computation by insect brains. Front. Neural Circuits 8:127. 10.3389/fncir.2014.00127 25389392 PMC4211400

[B27] Eichner H. Joesch M. Schnell B. Reiff D. F. Borst A. (2011). Internal structure of the fly elementary motion detector. Neuron 70, 1155–1164. 10.1016/j.neuron.2011.03.028 21689601

[B28] Fisher Y. E. Silies M. Clandinin T. R. (2015). Orientation selectivity sharpens motion detection in drosophila. Neuron 88, 390–402. 10.1016/j.neuron.2015.09.033 26456048 PMC4664581

[B29] Fitzgerald J. E. Clark D. A. (2015). Nonlinear circuits for naturalistic visual motion estimation. eLife 4:e09123. 10.7554/eLife.09123 26499494 PMC4663970

[B30] Fitzgerald J. E. Katsov A. Y. Clandinin T. R. Schnitzer M. J. (2011). Symmetries in stimulus statistics shape the form of visual motion estimators. Proc. Natl. Acad. Sci. U.S.A. 108, 12909–12914. 10.1073/pnas.1015680108 21768376 PMC3150910

[B31] Harrison R. R. Koch C. (1999). A robust analog VLSI motion sensor based on the visual system of the fly. Auton. Rob. 7, 211–224. 10.1023/A:1008916202887

[B32] Hassenstein B. Reichardt W. (1956). Systemtheoretische Analyse der Zeit-, Reihenfolgen- und Vorzeichenauswertung bei der Bewegungsperzeption des Rüsselkäfers Chlorophanus. Zeitschrift Für Naturforschung B 11, 513–524. 10.1515/znb-1956-9-1004

[B33] Hateren J. H. Schilstra C. (1999). Blowfly flight and optic flow. II. Head movements during flight. J. Exp. Biol. 202, 1491–1500. 10229695 10.1242/jeb.202.11.1491

[B34] Hennig P. Egelhaaf M. (2012). Neuronal encoding of object and distance information: a model simulation study on naturalistic optic flow processing. Front. Neural Circuits 6:14. 10.3389/fncir.2012.00014 22461769 PMC3309705

[B35] James A. (1992). Nonlinear operator network models of processing in the fly lamina, in Nonlinear Vision: Determination of Neural Receptive Fields, Function, and Networks, eds Pinter R. Nabet B. (Boca Raton, FL: CRC Press), 39–72.

[B36] Juusola M. (1995). Transfer of graded potentials at the photoreceptor-interneuron synapse. J. Gen. Physiol. 105, 117–148. 10.1085/jgp.105.1.117 7537323 PMC2216927

[B37] Juusola M. French A. S. Uusitalo R. O. Weckström M. (1996). Information processing by graded-potential transmission through tonically active synapses. Trends Neurosci. 19, 292–297. 10.1016/S0166-2236(96)10028-X 8799975

[B38] Juusola M. Weckström M. Uusitalo R. O. Korenberg M. J. French A. S. (1995). Nonlinear models of the first synapse in the light-adapted fly retina. J. Neurophysiol. 74, 2538–2547. 8747212 10.1152/jn.1995.74.6.2538

[B39] Kern R. Boeddeker N. Dittmar L. Egelhaaf M. (2012). Blowfly flight characteristics are shaped by environmental features and controlled by optic flow information. J. Exp. Biol. 215, 2501–2514. 10.1242/jeb.061713 22723490

[B40] Koenderink J. J. (1986). Optic flow. Vision Res. 26, 161–179. 10.1016/0042-6989(86)90078-7 3716209

[B41] Köhler T. Röchter F. Lindemann J. P. Möller R. (2009). Bio-inspired motion detection in an FPGA-based smart camera module. Bioinspir. Biomim. 4:015008. 10.1088/1748-3182/4/1/015008 19258686

[B42] Kral K. Poteser M. (1997). Motion parallax as a source of distance information in locusts and mantids. J. Insect Behav. 10, 145–163. 10.1007/BF02765480

[B43] Kress D. van Bokhorst E. Lentink D. (2015). How lovebirds maneuver rapidly using super-fast head saccades and image feature stabilization. PLoS ONE 10:e0129287. 10.1371/journal.pone.0129287 26107413 PMC4481315

[B44] Laughlin S. (1981). A simple coding procedure enhances a neuron's information capacity. Zeitschrift Für Naturforschung C 36, 910–912. 7303823

[B45] Laughlin S. B. (1994). Matching coding, circuits, cells, and molecules to signals: general principles of retinal design in the fly's eye. Prog. Retin. Eye Res. 13, 165–196. 10.1016/1350-9462(94)90009-4

[B46] Laughlin S. B. Hardie R. C. (1978). Common strategies for light adaptation in the peripheral visual systems of fly and dragonfly. J. Comp. Physiol. 128, 319–340. 10.1007/BF00657606

[B47] Leonhardt A. Ammer G. Meier M. Serbe E. Bahl A. Borst A. (2016). Asymmetry of *Drosophila* ON and OFF motion detectors enhances real-world velocity estimation. Nat. Neurosci. 19, 706–715. 10.1038/nn.4262 26928063

[B48] Lindemann J. P. (2005). On the computations analyzing natural optic flow: quantitative model analysis of the blowfly motion vision pathway. J. Neurosci. 25, 6435–6448. 10.1523/JNEUROSCI.1132-05.2005 16000634 PMC6725274

[B49] Lipetz L. E. (1971). The relation of physiological and psychological aspects of sensory intensity, in Principles of Receptor Physiology, Number 1 in Handbook of Sensory Physiology, ed Loewenstein W. R. (New York, NY: Springer Berlin Heidelberg), 191–225. 10.1007/978-3-642-65063-5_6

[B50] Lucas B. D. Kanade T. (1981). An iterative image registration technique with an application to stereo vision, in International Joint Conference on Artificial Intelligence, Vol. 81 (Vancouver, BC), 674–679.

[B51] Mafrica S. Godiot S. Menouni M. Boyron M. Expert F. Juston R.. (2015). A bio-inspired analog silicon retina with Michaelis-Menten auto-adaptive pixels sensitive to small and large changes in light. Opt. Express 23, 5614–5635. 10.1364/OE.23.005614 25836794

[B52] Mauss A. S. Meier M. Serbe E. Borst A. (2014). Optogenetic and pharmacologic dissection of feedforward inhibition in drosophila motion vision. J. Neurosci. 34, 2254–2263. 10.1523/JNEUROSCI.3938-13.2014 24501364 PMC6608528

[B53] Meyer H. G. Bertrand O. J. Paskarbeit J. Lindemann J. P. Schneider A. Egelhaaf M. (2016). A bio-inspired model for visual collision avoidance on a hexapod walking robot, in Conference on Biomimetic and Biohybrid Systems (Edinburgh, UK: Springer), 167–178.

[B54] Meyer H. G. Lindemann J. P. Egelhaaf M. (2011). Pattern-dependent response modulations in motion-sensitive visual interneurons-a model study. PLoS ONE 6:e21488. 10.1371/journal.pone.0021488 21760894 PMC3132178

[B55] Mronz M. Lehmann F.-O. (2008). The free-flight response of Drosophila to motion of the visual environment. J. Exp. Biol. 211, 2026–2045. 10.1242/jeb.008268 18552291

[B56] Muijres F. T. Elzinga M. J. Iwasaki N. A. Dickinson M. H. (2015). Body saccades of Drosophila consist of stereotyped banked turns. J. Exp. Biol. 218, 864–875. 10.1242/jeb.114280 25657212

[B57] Naka K. I. Rushton W. A. H. (1966). S-potentials from colour units in the retina of fish (Cyprinidae). J. Physiol. 185, 536–555. 10.1113/jphysiol.1966.sp008001 5918058 PMC1395833

[B58] Normann R. A. Perlman I. (1979). Evaluating sensitivity changing mechanisms in light-adapted photoreceptors. Vision Res. 19, 391–394. 10.1016/0042-6989(79)90101-9 473607

[B59] O'Carroll D. C. Barnett P. D. Nordström K. (2011). Local and global responses of insect motion detectors to the spatial structure of natural scenes. J. Vis. 11, 20. 10.1167/11.14.20 22201615

[B60] Plett J. Bahl A. Buss M. Kühnlenz K. Borst A. (2012). Bio-inspired visual ego-rotation sensor for MAVs. Biol. Cybern. 106, 51–63. 10.1007/s00422-012-0478-6 22350507

[B61] Rajesh S. Straw A. O'Carroll D. C. Abbott D. (2005). Effect of spatial sampling on pattern noise in insect-based motion detection, in Smart Materials, Nano-, and Micro-Smart Systems, ed Al-Sarawi S. F. (Sydney, NSW: International Society for Optics and Photonics), 811–825.

[B62] Rasumov N. Baker M. Niven J. Laughlin S. (2011). Adaptation reduces sensitivity to save energy without information loss in the fly visual system. Proc. Physiol. Soc. 22:C07.

[B63] Reichardt W. Schöner G. Gilroy L. (1961). Autocorrelation, a principle for the evaluation of sensory information by the central nervous system, in Sensory Communication, ed Rosenblith W. A. (Cambridge, MA: MIT Press), 303–317.

[B64] Reiff D. F. Plett J. Mank M. Griesbeck O. Borst A. (2010). Visualizing retinotopic half-wave rectified input to the motion detection circuitry of drosophila. Nat. Neurosci. 13, 973–978. 10.1038/nn.2595 20622873

[B65] Sanes J. R. Zipursky S. L. (2010). Design principles of insect and vertebrate visual systems. Neuron 66, 15–36. 10.1016/j.neuron.2010.01.018 20399726 PMC2871012

[B66] Santen J. P. H. V. Sperling G. (1985). Elaborated Reichardt detectors. J. Opt. Soc. Am. A 2, 300–321. 10.1364/JOSAA.2.000300 3973763

[B67] Schilstra C. Hateren J. H. (1999). Blowfly flight and optic flow. I. Thorax kinematics and flight dynamics. J. Exp. Biol. 202, 1481–1490. 10229694 10.1242/jeb.202.11.1481

[B68] Schwegmann A. Lindemann J. P. Egelhaaf M. (2014a). Depth information in natural environments derived from optic flow by insect motion detection system: a model analysis. Front. Comput. Neurosci. 8:83. 10.3389/fncom.2014.00083 25136314 PMC4118023

[B69] Schwegmann A. Lindemann J. P. Egelhaaf M. (2014b). Temporal statistics of natural image sequences generated by movements with insect flight characteristics. PLoS ONE 9:e110386. 10.1371/journal.pone.0110386 25340761 PMC4207754

[B70] Schwegmann A. Lindemann J. P. Egelhaaf M. (2014c). Translational Sequences of Panoramic High Dynamic Range Images in Natural Environments. Bielefeld: Bielefeld University Open Data Publication 10.4119/unibi/2689483

[B71] Shoemaker P. A. O'Carroll D. C. Straw A. D. (2005). Velocity constancy and models for wide-field visual motion detection in insects. Biol. Cybern. 93, 275–287. 10.1007/s00422-005-0007-y 16151841

[B72] Silies M. Gohl D. M. Clandinin T. R. (2014). Motion-detecting circuits in flies: coming into view. Annu. Rev. Neurosci. 37, 307–327. 10.1146/annurev-neuro-071013-013931 25032498

[B73] Silies M. Gohl D. M. Fisher Y. E. Freifeld L. Clark D. A. Clandinin T. R. (2013). Modular use of peripheral input channels tunes motion-detecting circuitry. Neuron 79, 111–127. 10.1016/j.neuron.2013.04.029 23849199 PMC3713415

[B74] Sobel E. C. (1990). The locust's use of motion parallax to measure distance. J. Comp. Physiol. A 167, 579–588. 10.1007/bf00192653 2074547

[B75] Straw A. D. Rainsford T. O'Carroll D. C. (2008). Contrast sensitivity of insect motion detectors to natural images. J. Vis. 8, 32–32. 10.1167/8.3.32 18484838

[B76] Strübbe S. Stürzl W. Egelhaaf M. (2015). Insect-inspired self-motion estimation with dense flow fields-an adaptive matched filter approach. PLoS ONE 10:e0128413. 10.1371/journal.pone.0128413 26308839 PMC4550262

[B77] Takemura A. Inoue Y. Gomi H. Kawato M. Kawano K. (2001). Change in neuronal firing patterns in the process of motor command generation for the ocular following response. J. Neurophysiol. 86, 1750–1763. 11600636 10.1152/jn.2001.86.4.1750

[B78] Tuthill J. C. Nern A. Rubin G. M. Reiser M. B. (2014). Wide-field feedback neurons dynamically tune early visual processing. Neuron 82, 887–895. 10.1016/j.neuron.2014.04.023 24853944

[B79] Vaina L. M. Beardsley S. A. Rushton S. K. (2004). Optic Flow and Beyond. Dordrecht; Boston, MA; London: Kluwer Academic Publishers.

[B80] van Hateren J. H. (1992a). Real and optimal neural images in early vision. Nature 360, 68–70. 1436076 10.1038/360068a0

[B81] van Hateren J. H. (1992b). Theoretical predictions of spatiotemporal receptive fields of fly LMCs, and experimental validation. J. Comp. Physiol. A 171, 157–170. 10.1007/BF00188924

[B82] van Hateren J. H. (1993). Spatiotemporal contrast sensitivity of early vision. Vision Res. 33, 257–267. 10.1016/0042-6989(93)90163-Q 8447098

[B83] van Hateren J. H. (1997). Processing of natural time series of intensities by the visual system of the blowfly. Vision Res. 37, 3407–3416. 10.1016/S0042-6989(97)00105-3 9425553

[B84] van Hateren J. H. Snippe H. P. (2001). Information theoretical evaluation of parametric models of gain control in blowfly photoreceptor cells. Vision Res. 41, 1851–1865. 10.1016/S0042-6989(01)00052-9 11369048

