# Supplementary material for: Peripheral Processing Facilitates Optic Flow-Based Depth Perception
Source: Front Comput Neurosci. 2016 Oct 21;10:111. doi: 10.3389/fncom.2016.00111 (PMC5073142; doi:10.3389/fncom.2016.00111)
Supplement: Supplementary file 1 [file Presentation1.PDF]

Supplementary Material: Peripheral processing facilitates optic flow-based depth perception

Jinglin Li *, **Jens P. Lindemann, and Martin Egelhaaf**
*Correspondence:
Jinglin Li
j.li@uni-bielefeld.de

1 SUPPLEMENTARY MOVIES

Movie S1. Essential peripheral computational units for optic flow-based spatial vision. Comparison of the representation of environmental parameters by arrays of different versions of motion detectors (pure EMD model (middle panel) and *PRelab1-LMCbasic-EMD* model (lower panel)) during translational flight in a forest (upper panel, panoramic visual input intensity sequences). For demonstration purposes, the movie is shown here only at a rate of 15 frames per second, while, in our simulations it was presented at a rate of 200 frames per second.

Movie S2. Robust spatial vision under a wide range of brightness conditions. Robust performance of the *PRelab1-LMCbasic-EMD* model version (lower panel) in representing contours of nearby objects under extremely dark (left) and extremely bright light conditions (right). The intensities during translational flight in a forest are artificially rescaled to the range of 10^2 to 10^4 (upper left) and 10^{10} to 10^{12} (upper right). For demonstration purposes, the movie is shown here only at a rate of 15 frames per second, while, in our simulations it was presented at a rate of 200 frames per second.

Movie S3. Role of spatial range of brightness adaptation. Comparison of the representation of environmental parameters by arrays of motion detectors (*PRelab1-LMCbasic-EMD* version) for global (middle panel) and local (lower panel) brightness adaptation. The light intensities vary over several decades (upper panel, with intensities artificially rescaled to the range of 10^1 to 10^6 in arbitrary units). For demonstration purposes, the movie is shown here only at a rate of 15 frames per second, while, in our simulations it was presented at a rate of 200 frames per second.

Movie S4. Time scale of brightness adaptation. Recovery of the representation of spatial information by arrays of motion detectors (*PRelab1-LMCbasic-EMD* version) after an abrupt change in light intensity (upper panel, 100 ms before and 690 ms after abrupt intensity change from the range of 10^2 to 10^4 to the range of 10^4 to 10^6) with default parameter settings (middle panel, τ_{PRlp1} = 9 ms, τ_{PRlp2} = 250 ms) and fast-adaptive parameter settings (lower panel, τ_{PRlp1} = 2 ms, τ_{PRlp2} = 20 ms). For demonstration purposes, the movie is shown here only at a rate of 15 frames per second, while, in our simulations it was presented at a rate of 200 frames per second.
